# A comprehensive gene-centric pleiotropic association analysis for 14 psychiatric disorders with GWAS summary statistics

**DOI:** 10.1186/s12916-021-02186-z

**Published:** 2021-12-13

**Authors:** Haojie Lu, Jiahao Qiao, Zhonghe Shao, Ting Wang, Shuiping Huang, Ping Zeng

**Affiliations:** 1 Department of Biostatistics, School of Public Health, Xuzhou Medical University, Xuzhou, 221004 Jiangsu China; 2 Center for Medical Statistics and Data Analysis, Xuzhou Medical University, Xuzhou, 221004 Jiangsu China; 3 Key Laboratory of Human Genetics and Environmental Medicine, Xuzhou Medical University, Xuzhou, 221004 Jiangsu China

**Keywords:** Psychiatric disorder, Pleiotropy, Genetic correlation, Gene-based association analysis, Genome-wide association study, Summary statistics, Pleiotropic analysis under composite null hypothesis, Mendelian randomization, Causal inference, Instrumental variable

## Abstract

**Background:**

Recent genome-wide association studies (GWASs) have revealed the polygenic nature of psychiatric disorders and discovered a few of single-nucleotide polymorphisms (SNPs) associated with multiple psychiatric disorders. However, the extent and pattern of pleiotropy among distinct psychiatric disorders remain not completely clear.

**Methods:**

We analyzed 14 psychiatric disorders using summary statistics available from the largest GWASs by far. We first applied the cross-trait linkage disequilibrium score regression (LDSC) to estimate genetic correlation between disorders. Then, we performed a gene-based pleiotropy analysis by first aggregating a set of SNP-level associations into a single gene-level association signal using MAGMA. From a methodological perspective, we viewed the identification of pleiotropic associations across the entire genome as a high-dimensional problem of composite null hypothesis testing and utilized a novel method called PLACO for pleiotropy mapping. We ultimately implemented functional analysis for identified pleiotropic genes and used Mendelian randomization for detecting causal association between these disorders.

**Results:**

We confirmed extensive genetic correlation among psychiatric disorders, based on which these disorders can be grouped into three diverse categories. We detected a large number of pleiotropic genes including 5884 associations and 2424 unique genes and found that differentially expressed pleiotropic genes were significantly enriched in pancreas, liver, heart, and brain, and that the biological process of these genes was remarkably enriched in regulating neurodevelopment, neurogenesis, and neuron differentiation, offering substantial evidence supporting the validity of identified pleiotropic loci. We further demonstrated that among all the identified pleiotropic genes there were 342 unique ones linked with 6353 drugs with drug-gene interaction which can be classified into distinct types including inhibitor, agonist, blocker, antagonist, and modulator. We also revealed causal associations among psychiatric disorders, indicating that genetic overlap and causality commonly drove the observed co-existence of these disorders.

**Conclusions:**

Our study is among the first large-scale effort to characterize gene-level pleiotropy among a greatly expanded set of psychiatric disorders and provides important insight into shared genetic etiology underlying these disorders. The findings would inform psychiatric nosology, identify potential neurobiological mechanisms predisposing to specific clinical presentations, and pave the way to effective drug targets for clinical treatment.

**Supplementary Information:**

The online version contains supplementary material available at 10.1186/s12916-021-02186-z.

## Background

In the past decade, twins studies and more recent genome-wide association studies (GWASs) have successfully identified a large amount of single-nucleotide polymorphisms (SNPs) that are robustly associated with diverse psychiatric disorders [1–5], revolutionizing our understanding of genetic architecture underlying these illnesses. Two major findings have been revealed. First, there exists strong evidence that psychiatric disorders are highly heritable and polygenic [1, 5–11], with the estimated heritability of 40–80% across a wide range of such disorders, which means that a large number of genes have weak effects and substantially contribute to disease risk. Second, a surprisingly high degree of genetic loci are found to exhibit significant effects on multiple clinically distinct psychiatric disorders [1, 3, 5–7, 11–13], a phenomenon which is well-known as pleiotropy and is also pervasively perceived for many other complex phenotypes [14–16].

Understanding the extent to which one psychiatric disorder shares similar genetic component with the others is critical for identifying the etiology of phenotypic relationships and can inform disease nosology and diagnostic practice and improve drug development [1, 3, 11–13, 17]. However, the shared genetic foundation among psychiatric disorders remains not fully understood and there also exist practical and statistical issues that need to be investigated further. First, previous studies only incorporated a small set of psychiatric disorders; thus, they cannot offer a systematic and complete viewpoint about the genetic connection among various disorders. Second, nearly all prior work focused mainly on SNP-level pleiotropy (Additional file 1: Table S1) [18–51]; the power of detecting single SNP association signal is still limited because genetic variants typically have weak effect on phenotypes [52–54]. Moreover, causal interpretation of SNP-based pleiotropic associations is challenging as truly causal genetic variants are often hard to pinpoint due to linkage disequilibrium (LD) among SNPs. Third, prior work primarily examined genetic correlation between psychiatric disorders [55]. Genetic correlation only quantifies an overall genetic similarity across the entire genome [56], it cannot characterize detailed association pattern for individual genetic loci and an insignificant estimate does not necessarily suggest the absence of common genetic background. Fourth, to identify commonly associated loci, almost all prior studies employed pleiotropy-informed mixture methods such as co-localization test [57], cFDR [58], GPA [20], and iMAP [59]. These methods were generally developed from a Bayesian perspective, their type I error rate control at a given family-wise error rate (FWER) is however not well established because they prioritize associations at a much more liberal significance level [60].

To overcome these limitations, in this work we attempt to address several critical issues in pleiotropy mapping for psychiatric disorders. First, instead considering individual SNPs, we implemented a gene-centric pleiotropy analysis by analyzing a set of SNPs located within a gene collectively. To this aim, relying on summary statistics of psychiatric disorders, we first conducted MAGMA [61] to aggregate a group of SNP-level association signals into a single gene-level association signal, based on which our pleiotropy analysis was carried out. Second, we analyzed a total of 14 psychiatric disorders, much larger compared to prior work; thus, our analysis had the potential to offer a comprehensive insight into shared genetic component underlying distinct disorders. Third, from a methodological perspective, we viewed the identification of pleiotropic gene associations across the whole genome as a high-dimensional problem of composite null hypothesis testing [62], and applied a powerful method called PLACO for pleiotropy mapping [60]. To further resolve the horizontal or vertical pleiotropy, we ultimately evaluated the potentially causal association among distinct psychiatric disorders using Mendelian randomization (MR) methods [63–68],

## Methods

### Summary statistics for 14 psychiatric disorders

We analyzed 14 psychiatric disorders obtained from the Psychiatric Genomics Consortium (PGC) (Table 1). These disorders included anorexia nervosa (AN; *N* = 14,477) [69], anxiety disorder (AD; *N* = 17,526) [70], autism spectrum disorder (ASD; *N* = 46,350) [71], alcohol use disorder (AUD; *N* = 121,604) [72], obsessive-compulsive disorder (OCD; *N* = 9725) [73], bipolar disorder (BIP; *N* = 51,710) [74], schizophrenia (SCZ; *N* = 77,096) [75], posttraumatic stress disorder (PTSD; *N* = 174,659) [76], Tourette’s syndrome (TS; *N* = 14,307) [77], cannabis use (CU; *N* = 184,765) [78], major depression disorder (MDD; *N* = 480,359) [79], and attention-deficit/hyperactivity disorder (ADHD; *N* = 53,293) [80]. For all the disorders, we obtained their European-only summary statistics and performed stringent quality control: (i) excluded non-biallelic SNPs and those with strand-ambiguous alleles; (ii) excluded SNPs that had no rs label; (iii) removed duplicated SNPs or those not included in the 1000 Genomes Project or whose alleles did not match those there; (iv) excluded SNPs that were located within the region of major histocompatibility complex (chr6: 28.5–33.5 Mb) because of its complex LD structure [81]; (v) kept SNPs that had minor allele frequency (MAF) > 0.01.

**Table 1:** Summary information of 14 psychiatric disorders analyzed in this study

Traits	*N* (case/control)	*m*	*S*	*λ*	Inter	*h*^2^ (se)	Reference
AD	17,526 (5761/11,765)	5,479,241	22,720	1.032	1.008	0.050 (0.025)	[70]
ADHD	53,293 (19,099/34,194)	6,414,003	22,583	1.213	1.043	0.191 (0.012)	[80]
AN	14,477 (3495/10,982)	6,639,324	22,610	1.219	1.056	0.124 (0.009)	[69]
ASD	46,350 (18,381/27,969)	7,076,650	22,783	1.132	1.013	0.141 (0.010)	[71]
BIP	51,710 (20,352/31,358)	7,479,414	23,031	1.143	1.017	0.288 (0.012)	[74]
CU	184,765 (53,180/131,585)	6,908,164	22,758	1.105	1.012	0.056 (0.003)	[78]
MDD	480,359 (135,458/344,901)	5,372,902	16,595	1.153	1.019	0.062 (0.003)	[79]
OCD	9725 (2688/7037)	7,138,930	22,645	1.237	1.004	0.252 (0.040)	[73]
PTSD	174,659 (23,212/151,447)	7,482,865	22,762	1.152	1.005	0.004 (0.002)	[76]
SCZ	77,096 (33,640/43,456)	7,684,282	23,135	1.030	0.985	0.357 (0.012)	[75]
TS	14,307 (4819/9488)	7,120,803	22,608	1.065	1.059	0.297 (0.032)	[77]
AUDIT-T	121,604	7,790,148	22,995	1.441	1.060	0.065 (0.004)	[72]
AUDIT-C	121,604	7,790,148	22,948	1.088	1.005	0.059 (0.004)	[72]
AUDIT-P	121,604	7,790,148	22,883	1.175	0.998	0.044 (0.004)	[72]

Note: *N* is the sample size of original GWASs; *m* is the number of SNPs used in MAGMA; *S* is the number of analyzed genes in MAGMA; *λ* is the genomic inflation factor estimated by LDSC; inter denotes the LDSC intercept; *h*^2^ is the SNP-based heritability estimated by LDSC. *AD* anxiety traits, *ADHD* attention-deficit/hyperactivity trait, *AN* anorexia nervosa, *ASD* autism spectrum trait, *AUDIT-T* alcohol use traits identification test based on total score, *AUDIT-C* alcohol use traits identification test based on consumption, *AUDIT-P* alcohol use traits identification test based on problematic consequences of drinking, *BIP* bipolar trait, *CU* cannabis use, *MDD* major depression trait, *OCD* obsessive-compulsive disorder, *PTSD* posttraumatic stress trait, *SCZ* schizophrenia, *TS* Tourette’s syndrome

### Estimate genetic correlation with LDSC

We first employed the cross-trait linkage disequilibrium score regression (LDSC) to assess the genetic correlation between two psychiatric disorders with genome-wide SNPs [81]. The LD score for every SNP was calculated based on genotypes of common SNPs (with MAF > 0.01 and the *P* value of the Hardy Weinberg equilibrium test> 1 × 10^−5^) with a 10 Mb window on 503 Europeans in the 1000 Genomes Project. Then, LDSC carried out a weighted linear model by regressing the product of *Z*-statistics of two traits on the LD score across all available genetic variants across the whole genome. Theoretically, the regression slope provides an unbiased estimate for genetic correlation even when overlapping individuals exist between the two GWASs. Based on estimated genetic correlations, we conducted a hierarchical cluster analysis for these disorders.

### Gene-based pleiotropic analysis under composite null hypothesis

To detect pleiotropic genes, we first applied MAGMA [61] to aggregate a set of SNP-level associations into a single gene-level association signal relying on summary statistics. It needs to emphasize that we used MAGMA here because it had been demonstrated that this method was powerful and computationally efficient and can be easily implemented with user-friendly software [61]. When conducting MAGMA, we defined the set of SNPs as those located within a given gene in terms of the annotation file provided in VAGIS [82]. Then, the *P* value of each gene was obtained and converted immediately into *Z* statistic. The direction of *Z* statistic was determined by the sign of summation of the product of effect sizes and MAFs of all SNPs in each gene [83]. Finally, depending on these newly transformed *Z* statistics, we carried out the pleiotropy test via PLACO [60], which was recently developed for detecting SNP-level pleiotropy by borrowing the perspective of composite null hypothesis from high-dimensional mediation analysis [62, 83]. We here extended it to discover pleiotropic associations at the gene level. Prior simulations [60] and variance-component-based mediation analysis under composite null hypothesis [84] already implied the validity of such extension. In brief, PLACO examines one gene at a time with two sets of *Z*-statistics as input and proceeds by dividing the composite null hypothesis of pleiotropy into three sub-null scenarios: (i) *H*_00_: the gene is not associated neither of the two disorders. (ii) *H*_10_: the gene is associated with the first disorder but not the second. (iii) *H*_01_: the gene is not associated with the first disorder but the second. The alternative hypothesis (*H*_11_) is that the gene is related to both disorders, corresponding to pleiotropic association.

The *P* values of MAGMA and PLACO were corrected by false discovery rate (FDR). Besides PLACO, we also leveraged a likelihood-ratio-based test (LRT) method to examine the existence of an overall pleiotropy between two disorders [20]. Moreover, as an empirical comparison, we performed two additional naïve methods. First, after obtaining *P* values for each disorder with MAGMA, we directly employed the FDR procedure to separately detect significant genes in each pair and identified genes having pleiotropic effect as those shared by both disorders; we referred to it as the direct FDR method. Second, we aggregated the two *P* values of a pair of disorders into a single *P* value by taking the maximum value, based on which pleiotropic genes were identified via the FDR procedure; we referred to it as the maximum *P* value method.

For each pleiotropic gene detected by PLACO, we simply calculated Pearson’s correlation coefficient (*r*) of SNP effect sizes to evaluate the similarity of genetic influence. We also assessed the effect heterogeneity of each SNP located within a pleiotropic gene through Cochran’s Q test, with the *P* value of heterogeneity corrected via the Benjamini-Yekutieli method to take the local dependency of SNPs into consideration [85].

### Functional analysis for pleiotropic genes

Afterwards, we performed differential expression analysis and gene set enrichment analysis for pleiotropic genes identified by PLACO using FUMA [86]. Gene expressions of 53 tissues were obtained from GTEx, and a total of 22,146 were finally considered. To obtain differentially expressed gene (DEG) sets for every tissue, expressions were first normalized and then analyzed with the two-sided Student’s *t* test for each gene in one tissue against all others. Genes with Bonferroni-corrected *P* < 0.05 and absolute log-fold change ≥ 0.58 were defined as a DEG set in a given tissue [87–90], indicating that expression levels of these genes in that tissue had larger discrepancy compared to those in others. Upregulated and downregulated genes were further distinguished in a tissue by taking the sign of *t*-score into account. Finally, pleiotropic genes were tested against those DEG sets by hyper-geometric tests to evaluate whether an overrepresentation existed in DEG sets for special tissues.

To assess an overrepresentation of biological functions in the gene set enrichment analysis, we examined these detected pleiotropic genes against gene sets obtained from MsigDB (i.e., hallmark gene sets, positional gene sets, curated gene sets, motif gene sets, computational gene sets, GO gene sets, oncogenic signatures, and immunologic signatures) and WikiPathways using hyper-geometric tests [86]. The correction for multiple comparisons was performed per data source of tested gene sets (e.g., canonical pathways, GO biological processes, and hallmark genes) using FDR. FUMA reported gene sets with adjusted *P* ≤ 0.05 and the number of genes that overlapped with the gene set > 1 by default. For all identified pleiotropic genes, we also investigated potential antagonistic or shared drug-gene interactions related to psychiatric disorders by exploring the DGIdb database [91, 92].

### Causal association among psychiatric disorders inferred via MR

MR is a commonly used instrumental variable causal inference for investigating the exposure on outcome effect with exposure-associated SNPs serving as instruments [63–68] (Additional file 2: Fig. S1). It is worth emphasizing that MR would not only offer an in-depth insight into the causal connection between these disorders, but also would provide a meaningfully genetic interpretation regarding to the nature of comorbidity of these disorders by resolving the horizontal or mediated (or vertical) pleiotropy [1, 71, 80] (Additional file 2: Fig. S2). We here conducted two major MR analyses. First, we aimed to study whether a childhood-onset psychiatric disorder (e.g., ASD or ADHD) would causally affect adulthood-onset psychiatric disorders (e.g., AD, AN, AUD, OCD, BIP, SCZ, PTSD, TS, CU, and MDD). To this aim, we carried out a one-sided MR analysis with ASD or ADHD as the exposure and adulthood-onset psychiatric disorders as outcomes. Second, because the temporal ordering among adulthood-onset disorders is not completely definitive, we intended to explore whether adulthood-onset psychiatric disorders may causally impact with each other. To this goal, we implemented a bidirectional MR analysis with one adulthood-onset disorder as the exposure and the remaining as outcomes. Because of the existence of high genetic overlap among the three alcohol use disorders, we only analyzed AUDIT-C in the two MR analyses (actually, AUDIT-T and AUDIT-P generated very similar results).

We selected SNP instruments using the clumping procedure of PLINK following prior work [93, 94]. During the clumping selection, we set the LD and physical distance thresholds to be 0.001 and 10 Mb, respectively, with LD estimated using a reference panel of 503 individuals of European ancestry in the 1000 Genomes Project. More importantly, as some psychiatric disorders had only a few independent genome-wide significant genetic loci (*P* < 5 × 10^−8^), to obtain sufficient SNPs serving as candidate instruments for fair comparison across diverse disorders, we employed a relatively relaxed significance cutoff of 1 × 10^−5^ for choosing associated genetic variants as done in [78]. In practical MR analysis, smaller significance threshold was often applied when few SNPs were available for the exposure at a more stringent level [95–97]. This would certainly generate a larger set of instruments that can thus explain larger phenotypic variation for power improvement; however, it also increased the potential risk of horizontal pleiotropy (Additional file 2: Fig. S1). Therefore, to avoid such issue, we additionally conducted a conservative quality control on candidate instruments by filtering out SNPs that might be potentially associated with the disorder under analysis if the selected SNP instruments had a Bonferroni-corrected *P* < 0.05 for that disorder [67, 98–100]; doing this would also minimize the influence of linkage association on our MR results [101].

In our MR study, we primarily employed the inverse-variance weighted (IVW) methods to estimate the causal effect [102–105]. To assess the robustness of significant associations identified by the IVW approach, we further undertook two complementary sensitivity analyses: (i) the weighted median-based method which is appropriate when some SNP instrumental variables are likely invalid [106] and (ii) the MR-Egger regression for which the intercept can be used to evaluate the directional pleiotropy of instruments [104, 107].

## Results

### Estimated cross-trait genetic correlation and cluster analysis

**Fig. 1:**
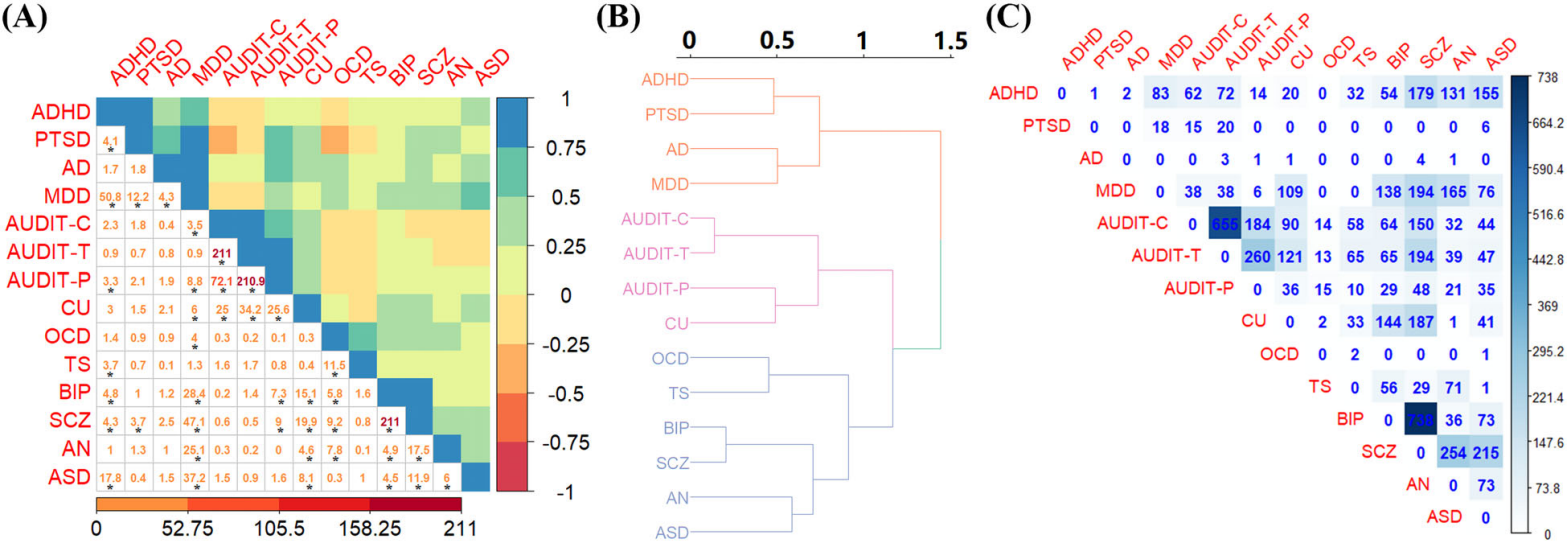
**A** Estimated genetic correlation of 14 psychiatric disorders with the LDSC method. The color on the top triangle indicates the magnitude of the genetic correlation; the significance of genetic correlation in − log10(*P* value) is shown on the bottom triangle, with significant genetic correlations after Bonferroni correction marked with an asterisk. **B** Cluster analysis based on the estimated genetic correlation matrix produced from cross-trait LDSC for the 14 psychiatric disorders. **C** Number of pleiotropic genes (FDR < 0.05) discovered by PLACO based on de-correlated *Z*-statistics for the 14 psychiatric disorders

We first present the result of estimated cross-trait genetic correlation (Fig. 1A). It is shown more than half of (75.8% = 69/91) pairs of psychiatric disorders exhibit positive genetic correlation, with an average of 0.219 and individual correlation coefficients ranging from − 0.354 ± 0.146 between AUDIT-C and PTSD to 0.986 ± 0.002 between AUDIT-C and AUDIT-T. Approximately 64.8% of these genetic correlation estimates have *P* values< 0.05 and 44.0% are still significant after Bonferroni’s correction. It needs to highlight that the genetic correlation analysis for the three AUD traits might be biased because of overlapping samples although LDSC takes such issue into account [81].

Moreover, in terms of the cluster analysis with estimated genetic correlations, these psychiatric disorders can be genetically divided into three major categories (Fig. 1B). In brief, the first category consists primarily of disorders characterized by compulsive behaviors (e.g., BIP, SCZ, ASD, AN, OCD, and TS); the second factor is characterized by substance behavioral traits (e.g., AUDIT-P, AUDIT-T, AUDIT-C, and CU); the third factor is mainly characterized by depression and stress behaviors (e.g., ADHD, PTSD, AD, and MDD). Overall, the genetic correlation analysis indicates the existence of substantial common genetic basis across diverse psychiatric disorders.

### Shared associated genes for 14 psychiatric disorders

The direct FDR method discovers 0.18% of genes showing pleiotropic effect, the maximum *P* value method detects 0.05% of genes displaying pleiotropic impact (Additional file 2: Fig. S3), whereas PLACO identifies 0.31% of genes exhibiting pleiotropic influence. By leveraging LRT, we find strongly statistical evidence supporting shared genetic foundation underlying most of pairs (64.8%) of psychiatric disorders (Additional file 2: Fig. S4). Totally, there are 5884 associations (2424 unique genes) (FDR < 0.05) detected by PLACO across all pairs of psychiatric disorders (Fig. 1C and Additional file 3: Table S2). Among these disorders, we discover that SCZ shares the most pleiotropic associations with BIP (i.e., 738 shared genes), in line with the high genetic correlation between them (*r*_*g*_ = 0.85 ± 0.02) and also consistent with previous observation that extensively common polygenic variation contributes to the risk of the two disorders [1, 9, 55, 108, 109]. Interestingly, SCZ also shares a large number of pleiotropic genes with the three AUD traits (i.e., 150 with AUDIT-C, 48 with AUDIT-P, and 194 with AUDIT-T) although their genetic correlations are not evidently high (*r*_*g*_ = − 0.03 ± 0.02 with AUDIT-C, *r*_*g*_ = 0.18 ± 0.03 with AUDIT-P, and *r*_*g*_ = 0.02 ± 0.02 with AUDIT-T). Moreover, we find the number of identified pleiotropic genes is slightly positively correlated to the effective sample size (*r* = 0.181) and strongly positively correlated to the estimated heritability (*r* = 0.548) across all analyzed psychiatric disorders.

#### Correlation of effect sizes and heterogeneity of SNPs for pleiotropic genes

**Fig. 2:**
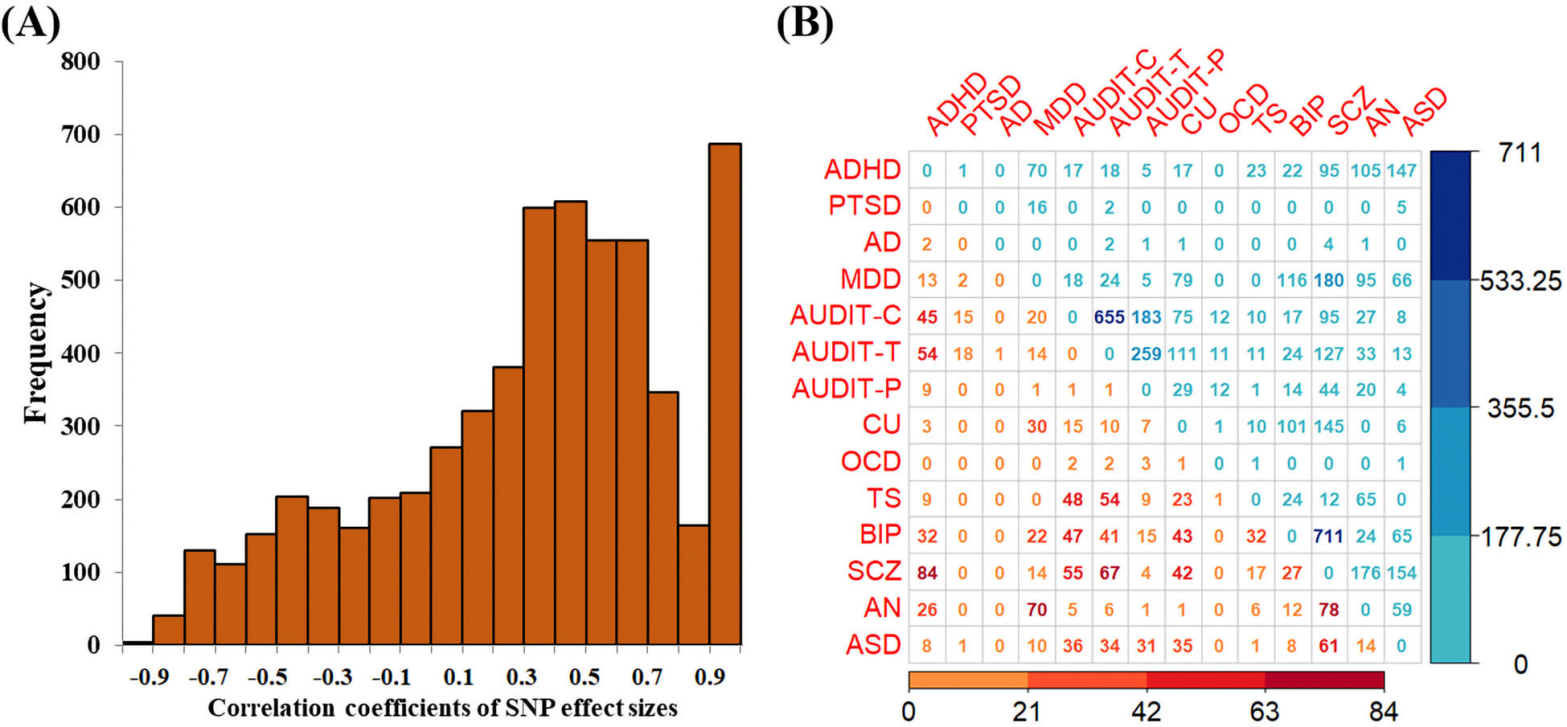
**A** Distribution of correlation coefficient of SNP effect sizes of pleiotropic genes detected by PLACO. **B** Number of pleiotropic genes having positive (the upper triangular) or negative (the lower triangular) correlation in SNP effect sizes on psychiatric disorders

We show estimated Pearson’s correlation coefficients of SNP effect sizes for each pleiotropic gene in a pair of psychiatric disorders in Fig. 2A. It is found most of pleiotropic genes (76.2%) exhibit positively correlated genetic effects, with an average of *r* = 0.529 (Fig. 2B), indicating that the majority of these genes generally show consistent direction in genetic influence on psychiatric disorders. Particularly, 39.2% of pleiotropic genes display substantially positive correlation in genetic effect (|*r*| > 0.5) and 11.7% show very strongly positive correlation (|*r*| > 0.9). Nevertheless, 23.8% of pleiotropic genes display negatively correlated genetic effects, with an average of *r* = − 0.377 (Fig. 2B), which implies that diverse functional roles of these genes underlie the pathological mechanism of psychiatric disorders and that the overall genetic correlation described above might be underestimated the genetic overlap among these disorders. Note that, the antagonistic effect phenomenon is also widely observed in other traits such as immune-relevant diseases [108, 110, 111].

On the other hand, in terms of the Cochran’s Q test, we discover on average 13.1% (ranging from 0% between AD and AN to 51.8% between AD and ADHD) of these SNPs show heterogeneous genetic effect (FDR < 0.05) (Additional file 2: Fig. S5A). As expected, the average proportion of heterogeneous SNPs across identified pleiotropic genes in a pair of psychiatric disorders is significantly inversely correlated to the cross-trait genetic correlation (*r* = − 0.32, *P* = 5.93 × 10^−3^; Additional file 2: Fig. S5B), suggesting the heterogeneity in genetic influence may partly explain the discrepancy of symptoms for these psychiatric disorders.

#### Pleiotropic genes associated with multiple psychiatric disorders

**Fig. 3:**
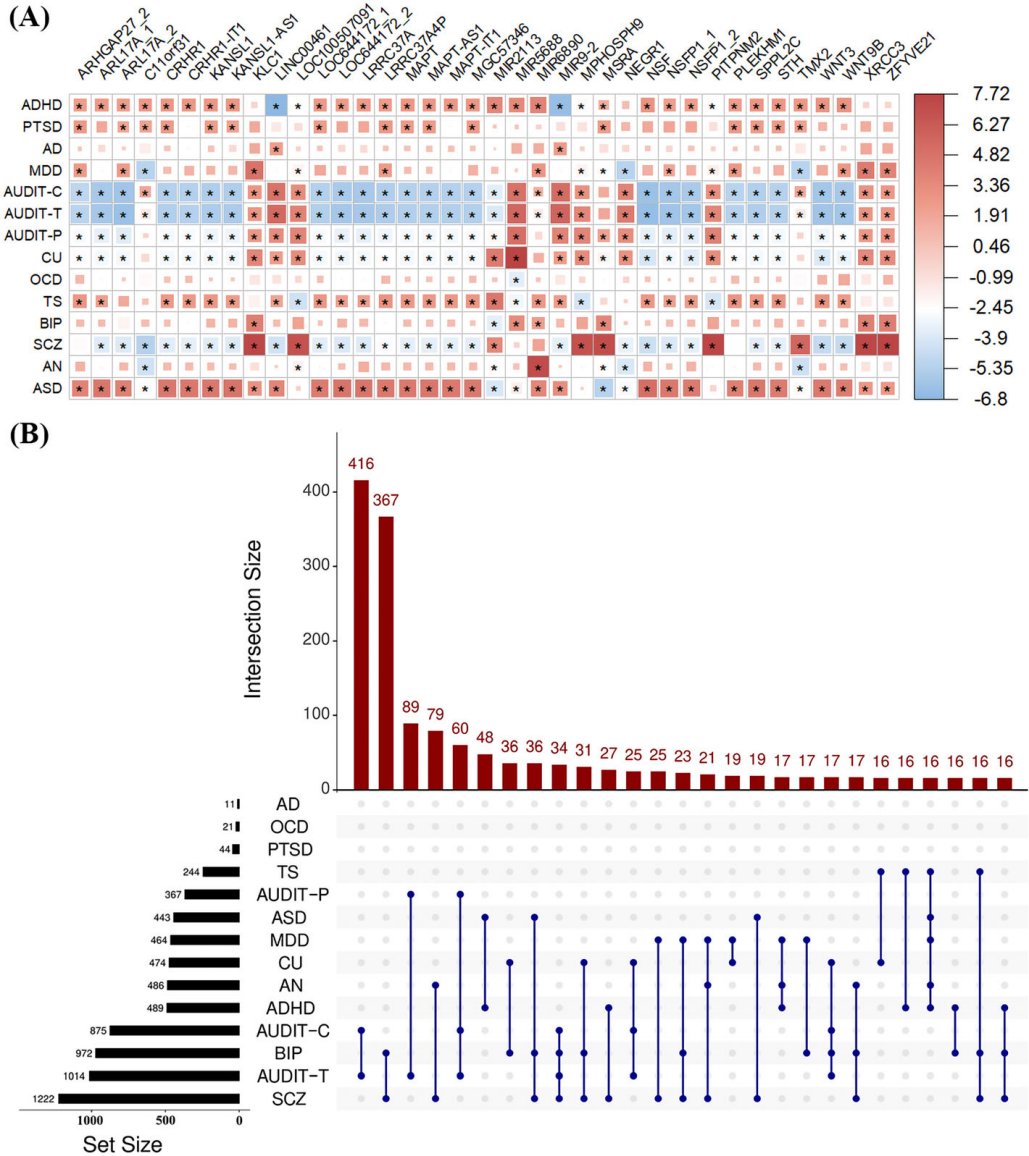
**A** Several associated genes which are shared across psychiatric disorders and are identified to show pleiotropic effects on at least eight psychiatric disorders. Color indicates direction and strength of associations across disorders. **B** UpSet plot to illustrate the numbers (*N* > 15) and distribution of pleiotropic genes shared across psychiatric disorders and the number of pleiotropic genes in each psychiatric disorder

Among all these pleiotropic genes, 44.0% are associated with at least three psychiatric disorders (Fig. 3A and Additional file 4: Table S3). The numbers and distribution of pleiotropic genes shared across disorders are demonstrated in Fig. 3B. Particularly, *LRRC37A4P* and *MIR2113* are the most top genes that are identified in 10 psychiatric disorders, followed by *LINC00461*, *MIR9-2*, *ARHGAP27_2*, *ARL17A_2*, *CRHR1*, *KANSL1*, *KANSL1-AS1*, *LOC100507091*, *LOC644172_1*, *MAPT*, *MAPT-AS1*, *MGC57346*, *MIR5688*, *MSRA*, *NSFP1_1*, *PLEKHM1*, *SPPL2C*, *STH*, and *WNT9B*, which are detected in 9 psychiatric disorders. *MIR2113* (microRNA 2113), on chromosome 6q16 [112, 113], was reportedly related to bipolar disease through being involved in multiple biological pathways that regulated brain development and synaptic plasticity [114]. In terms of two prospective longitudinal cohort studies, the gene *CRHR1* was suggested to exert a protective effect against adult depression among subjects who reported childhood maltreatment by consolidating memories of emotionally arousing experiences [115]. The deficiency of *KANSL1* can lead to neuronal dysfunction by oxidative stress-induced autophagy [116] and psychiatric symptoms were relatively common in *MAPT* mutation non-carriers compared to the general population [117]. The gene *MGC57346* was recently identified to be associated with neuroticism that was a common brain-related disorder [118]. In addition, *WNT9B* might be involved in the development of ASD via the WNT pathway [119]. Again, it is shown that these pleiotropic genes also exhibit antagonistic effects although they generally show similar genetic impacts in the same direction across psychiatric disorders (Fig. 3A).

#### Enrichment analysis for identified pleiotropic genes

**Fig. 4:**
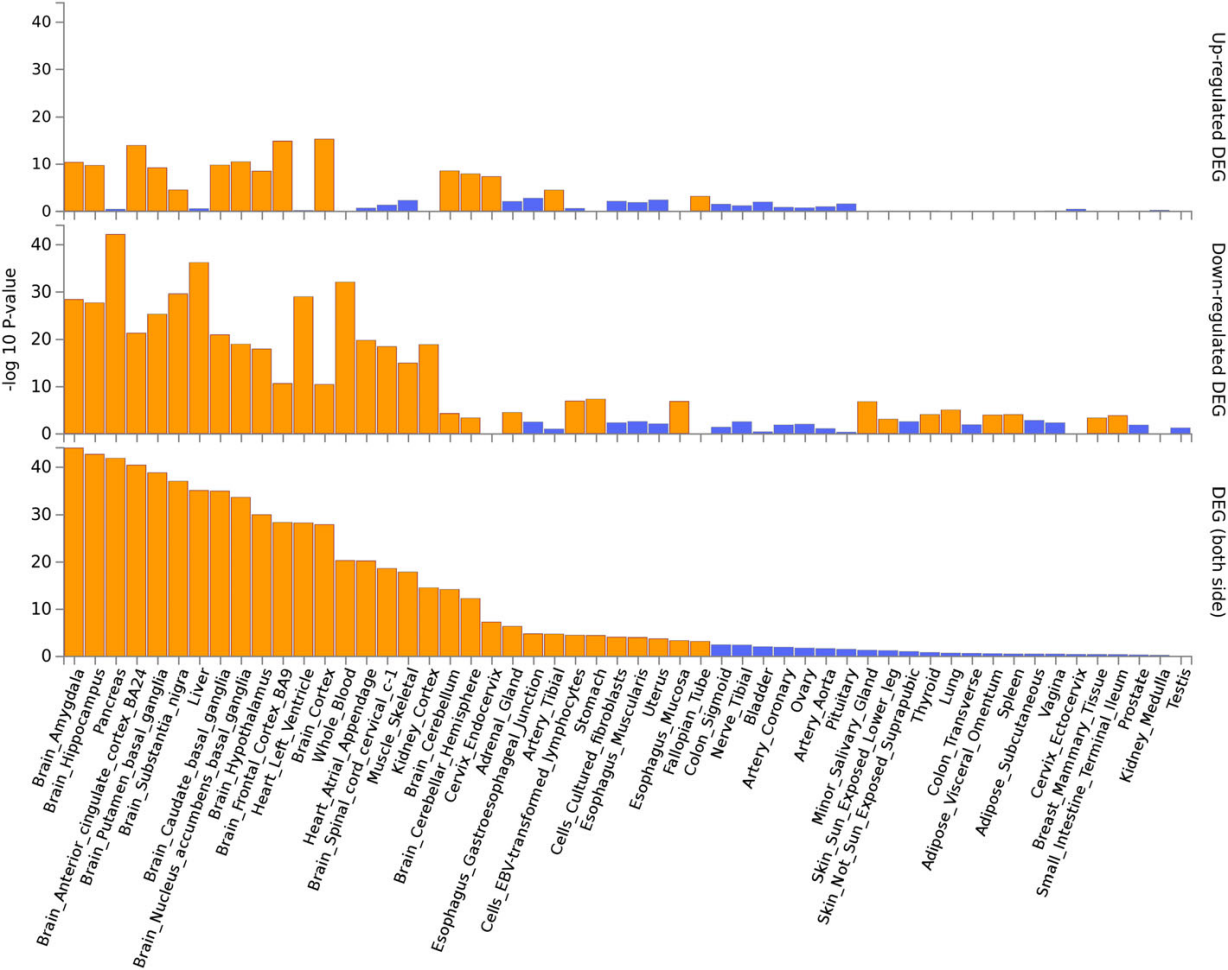
Enrichment of differentially expressed ones of all identified pleiotropic genes in terms of expression level across 54 GTEx tissues. *P* values are shown in the *y*-axis with a scale of − log10. The bars in orange represent significant enrichment with Bonferroni adjustment for multiple hypothesis testing

**Fig. 5:**
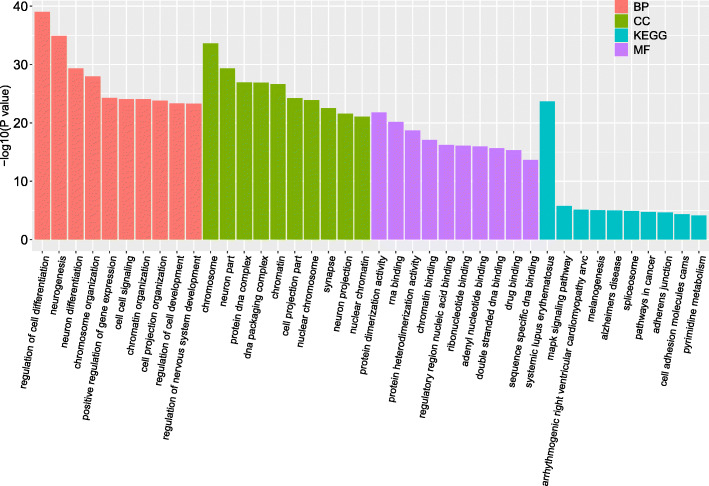
Top 10 significant types of pathways in terms of the GO and KEGG enrichment analyses. BP: biological process; CC: cellular component; MF: molecular function; KEGG: KEGG pathways

We here performed gene set enrichment analyses for all the 2424 unique pleiotropic genes identified by PLACO using FUMA. It is shown that the differentially expressed ones of these pleiotropic genes are significantly enriched in pancreas, liver, heart, and brain tissues in terms of expression level across the GTEx tissues, particularly for these downregulated pleiotropic genes (Fig. 4). The gene ontology (GO) enrichment analysis shows that the biological process (BP) of these pleiotropic genes is remarkably enriched in regulating neurodevelopment, such as cell differentiation (FDR = 7.27 × 10^−36^), neurogenesis (FDR = 4.60 × 10^−32^), and neuron differentiation (FDR = 1.08 × 10^−26^). For the GO cellular component (CC) terms, these genes are concentrated in chromosome (FDR = 2.32 × 10^−31^), neuron part (FDR = 2.29 × 10^−27^), and protein DNA complex (FDR = 3.27 × 10^−25^). For the molecular function (MF) categories, these genes are enriched in protein dimerization activity (FDR = 2.57 × 10^−19^), RNA binding (FDR = 5.62 × 10^−18^), and protein heterodimerization activity (FDR = 1.13 × 10^−16^). Meanwhile, the KEGG enrichment analysis shows that these genes are remarkably enriched in systemic lupus erythematosus (FDR = 3.77 × 10^−22^), MAPK signaling pathway (FDR = 1.67 × 10^−4^), and arrhythmogenic right ventricular cardiomyopathy ARVC (FDR = 3.88 × 10^−4^). The top 10 significant GO and KEGG pathways are shown in Fig. 5. Overall, these enrichment results further support the validity of these identified pleiotropic genes.

#### Investigation of drug-gene interactions for psychiatric disorders

It is demonstrated that among all the analyzed pleiotropic genes there are 342 unique ones which are associated with 6353 drugs showing drug-gene interaction with psychiatric disorders. However, only two genes (i.e., *HSP90B1* and *NCAM1*) show the same directional effects across all the 14 psychiatric disorders and the others display opposite directional effects on two or more disorders (Additional file 5: Table S4). Particularly, *CYP2D6* is the top gene related to 589 drugs, followed by *VDR* (460 drugs), *KDM4A* (442 drugs), *MAPT* (436 drugs), *FEN1* (199 drugs), *HTT* (198 drugs), *EGFR* (178 drugs), *DRD2* (177 drugs), and *MAPK1* (161 drugs). These drugs can be classified into distinct types including inhibitor (11.2% = 710/6353), agonist (3.6% = 228/6353), blocker (3.4% = 214/6353), antagonist (3.3% = 208/6353), and modulator (1.1% = 68/6353). With regard to these discovered gene-drug interactions, some prior studies provided evidence supporting their link with psychiatric disorders. For instance, *CYP2D6* played an important role in the efficacy of imipramine, clomipramine, nortriptyline, and fluoxetine in depressed patients [120–122]. As another example, it was shown that, by regulating the well-known tau protein in neuronal axons, *MAPT* interacted actively with astemizole and lansoprazole, two benzimidazole derivatives which were proven to have a great potential in the treatment of brain-related disorders [123, 124].

### Estimated causal associations between psychiatric disorders

**Fig. 6:**
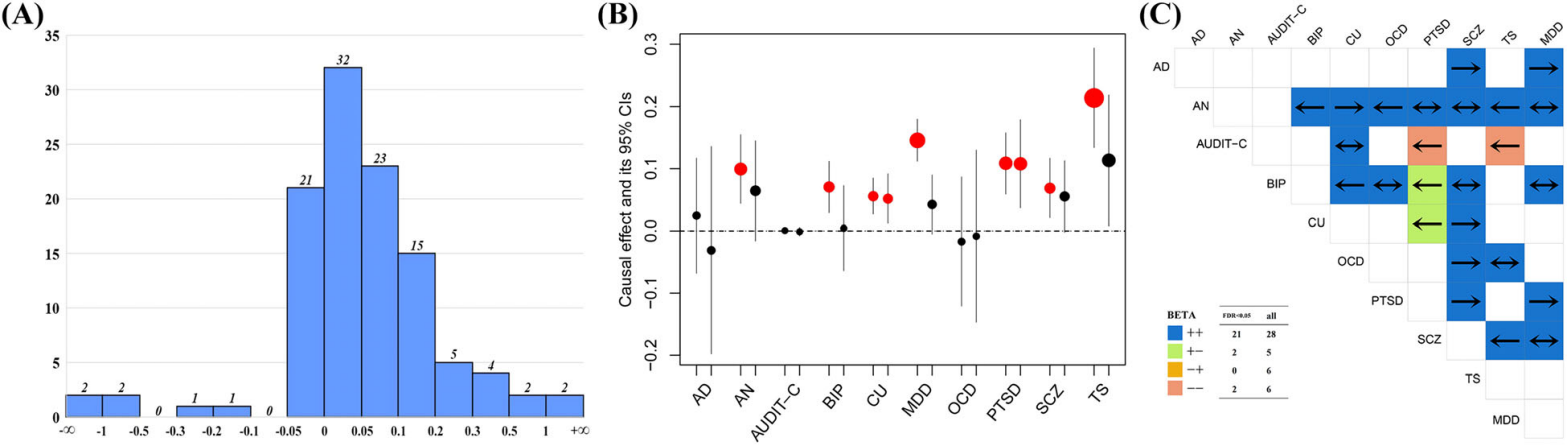
Results of the Mendelian randomization analysis for psychiatric disorders. **A** Distribution of effect sizes across all pairs of psychiatric disorders estimated with the inverse-variance weighted method. **B** Estimated effect sizes and their 95% confidence intervals (CIs) for two childhood-onset psychiatric disorders (e.g., ASD or ADHD) on ten adulthood-onset psychiatric disorders. **C** Significant causal associations among ten adulthood-onset psychiatric disorders indicated by arrows (FDR < 0.05). The arrow to the right side indicates that the adulthood-onset psychiatric disorders across the diagonal line has a significant causal effect on the adulthood-onset psychiatric disorders on the column, vice versa for the arrow to the left side; the two-sided arrow indicate that the two disorders have a significant causal effect on each other. The color indicates the direction of the estimated causal effect no matter whether it is significant or not. The plus and minus signs indicate positive and negative effect sizes, respectively. The legend on the bottom left shows the count of diverse effect sizes in direction for all the pairwise relationships or only these significant associations for adulthood-onset psychiatric disorders

The number of SNP instruments used for psychiatric disorders ranges from 19 to 433, with a median of 66. On average, the selected SNPs explain 4.3% of phenotypic variance across all psychiatric disorders. The minimum *F* statistic is above 10 (from 21.5 to 26.7) [102], indicating that weak instrumental bias is less likely to occur. We then present the results of the two MR analyses. First, among all the 110 examined relationships (20 for childhood-onset psychiatric disorders and 90 for adulthood-onset psychiatric disorders), most of the estimated effect sizes (whether significant or not) are positive (75.5% = 83/110) (Fig. 6A), with an average of 0.052 (sd = 0.352), implying the occurrence of one psychiatric disorder can lead to a substantially increased risk of other particular psychiatric disorders. Totally, there are 43 significant causal associations (FDR < 0.05) (i.e., 9 for childhood-onset psychiatric disorders and 34 for adulthood-onset psychiatric disorders). As anticipated, these significant relationships show higher average effect size compared to non-significant ones (0.110 vs. 0.015).

Second, we observe that the two childhood-onset psychiatric disorders can causally affect the development of psychiatric disorders in adulthood (Fig. 6B). For example, in terms of the estimated odds ratio (OR), both ADHD and ASD can similarly elevate the risk of CU (5.8% [95% confidence intervals (CIs) 2.6 ~ 8.9%] and 5.3% [95%CIs 1.2 ~ 9.6%] increase, respectively) and PTSD (11.5% [95%CIs 6.1 ~ 17.1%] and 11.4% [95%CIs 3.8 ~ 19.6%] increase, respectively). However, compared to ASD, ADHD seems to result in a higher risk for a much wider spectrum of adulthood-onset psychiatric disorders, including an increased risk of 10.5% (95%CIs 4.5~16.8%) for AN, 7.3% (95%CIs 2.9~11.9%) for BIP, 15.7% (95%CIs 11.8~19.7%) for MDD, 7.1% (95%CIs 2.1~12.4%) for SCZ, and 23.9% (95%CIs 14.3~34.2%) for TS. In addition, despite being grouped into the same cluster (see Fig. 1B), ASD does not causally affect any other psychiatric disorders that belong to the same category; in contrast, as described above, ADHD causally impacts two psychiatric disorders (e.g., PTSD and MDD but not AD) which belong to the same group.

Third, the average effect size is 0.112 ± 0.555 across all the 34 significant associations for these adulthood-onset psychiatric disorders, indicating that adulthood-onset psychiatric disorders themselves often strongly promote the disease development and progression with each other. More than half of them (61.8% = 21/34) exhibit bidirectionally positive influences on each other, this value slightly increases up to 62.2% (=28/45) for all pairwise relationships among adulthood-onset psychiatric disorders (Fig. 6C). For example, SCZ leads to a 51.4% (95%CIs 47.0 ~ 55.8%) increased risk of BIP, which can in turn increase the risk of SCZ by 44.0% (95%CIs 37.8~50.5%); as another example, AN results in a 9.1% (95%CIs 5.3~13.1%) higher risk of MDD, which also increase the risk of AN by 44.8% (95%CIs 26.7~65.5%). Note that not all of the bidirectionally positive effect sizes are statistically significant; for instance, SCZ can lead to a 6.8% (95%CIs 3.8~9.8%) higher risk of CU, but the impact of CU on SCZ is not significant (FDR = 0.270), which is consistent with the observation in [78]. In addition, only a few examined relationships show opposite effects (2 for significant associations and 5 for all pairwise relationships). Particularly, there are two significant relationships showing bidirectionally negative impacts; that is, alcohol use disorder is inversely affected by PTSD (OR = 0.29, 95%CIs 0.14~0.58, FDR = 0.002) and TS (OR = 0.13, 95%CIs 0.03~0.30, FDR = 0.024). However, neither PTSD nor TS has a significant effect on alcohol use although both have a negative effect (*β* = − 2.8 × 10^−3^, 95%CIs − 8.0 × 10^−3^~2.0 × 10^−3^, and FDR = 0.416 for PTSD; *β* = − 1.1 × 10^−3^, 95%CIs − 4.0 × 10^−3^~2.0 × 10^−3^, and FDR = 0.683 for TS).

Finally, for these significant associations described above, other MR methods generate very consistent results (Additional file 6: Table S5). For example, the estimates of causal effects obtained via the weighted median method are highly similar with those estimated with the IVW method in magnitude and direction (*r* = 0.973, *P* = 6.90 × 10^−28^), and all remain significant (FDR < 0.05), indicating the robustness of the IVW MR result. However, in terms of Egger regression, the majority of these associations are no longer significant although most of them show the same directional effects. This observation can be anticipated as Egger regression was developed under more limited conditions and is generally conservative [104, 107]. Moreover, in terms of the result of MR-Egger regression, we can largely rule out the potential influence of horizontal pleiotropy for most of these identified causal associations (with one exception for the association between BIP to SCZ) (i.e., the MR-Egger intercept is not significantly different from zero) (Additional file 6: Table S5).

## Discussion

### Summary of results in the present study

In the present work, we have carried out a systematic pleiotropy analysis for 14 psychiatric disorders, encompassing approximately 1.3 million cases and controls of European ancestry. Relying on GWAS summary statistics and applying a set of novel bioinformatics approaches, our analysis provides important insight into genetic background underlying these disorders. First of all, we reinforced the high heritability and the existence of widely common genetic component among psychiatric disorders at the whole genomic level [1, 3, 5–7, 11–13], which leads to the hypothesis that these disorders may be an extreme manifestation of continuous heritable traits, and also in part offers a reasonable explanation for the comorbidity observed in epidemiological studies [125, 126]. Although a few genetic correlations were non-significant, we cannot completely rule out the possibility that the null genetic correlations may be due to large estimation uncertainty for some psychiatric disorders because of small sample sizes (e.g., *N* is only 9725 for OCD).

Moreover, based on estimated genetic correlations, we found that these psychiatric disorders can be clustered into several distinct sub-groups, which, together with the high genetic overlap among these disorder, challenges the biological validity of existing diagnostic approaches that primarily rely on expert opinions, subjective description and experience of patients, and observational and syndromic systems of diagnosis and classification for psychiatric disorders [11]. This genetic overlap also offers a potentially alternative nosology informed by the similarity of disease genetic architecture underlying these disorders besides clinical manifestations [3, 11]. By carrying out the pleiotropy association analysis with PLACO [60], we detected a large number of potential pleiotropic genes for these psychiatric diseases. Furthermore, using the MR method we discovered, there existed a wide range of substantially causal associations between childhood-onset and adulthood-onset psychiatric disorders as well as among adulthood-onset psychiatric disorders, indicating that these disorders can actually cause each other and that genetic overlap and causality may commonly drive the observed co-existence of psychiatric disorders [11, 78].

### Comparison our discoveries to prior studies

#### Different statistical perspective and pleiotropy test method

Compared to prior pleiotropy studies of psychiatric disorders that mainly focused on individual SNPs (Additional file 1: Table S1) [18–51], our work has two pronounced features. First, we performed a gene-centric analysis based on a set of local SNPs rather than individual genetic variants. It is well known that gene is a more biologically meaningful functional unit in living organisms and a gene typically contains multiple association signals. Therefore, as an effective alternative analysis strategy, SNP-set analysis is in general more powerful than its counterpart of single-marker analysis due to the aggregation of multiple weak association signals and the reduced burden of multiple testing [127–134]. Second, we explicitly addressed the problem of pleiotropy identification from a statistical perspective of composite null hypothesis and applied PLACO to detect genes with pleiotropic effects [60]. Compared to previous methods whose error rate control for FWER was not well studied, PLACO was demonstrated to have well-calibrated error control and behaved better in power compared to other existing methods. Importantly, PLACO can be still valid even when overlapping subjects exist between diverse GWASs [60], which is not uncommon in large-scale meta-GWASs for phenotypic correlated traits. For example, it is shown there are about 2% of cases overlapped among these PGC GWASs for psychiatric disorders [1]. Note that, overlapping subjects can inflate test statistics of association signals [135–137]. Therefore, our pleiotropy analysis implemented with PLACO is less likely biased by overlapping subjects.

#### Comparison of estimated genetic correlation

It is worth highlighting that our estimates of genetic correlation based on summary-level data are largely consistent with those obtained with individual-level genotypes and phenotypes [55]. For example, both showed that SCZ and BIP shared a greatly high degree of genetic basis and revealed the existence of substantial genetic overlap between SCZ and MDD, SCZ and ASD, BIP and MDD, and MDD and ADHD. Nevertheless, opposite results are observed; for instance, we discovered a substantial positive genetic correlation between ASD and ADHD (*r*_*g*_ = 0.40, *P* = 1.44 × 10^−18^), which is supported by the observation that the two disorders co-occur with each other [138], in contrast to a negative but non-significant value observed in prior work (*r*_*g*_ = − 0.13, *P* = 0.13) [55]. Further, we discovered there existed a significantly positive genetic correlation between BIP and ADHD (*r*_*g*_ = 0.17, *P* = 1.75 × 10^−5^), unlike the lack of genetic overlap between them (*r*_*g*_ = 0.05, *P* = 0.31) discovered in prior study [55]. These evident distinctions imply the advantage and benefit of our analysis using much larger sample sizes for these psychiatric disorders.

#### Comparison of genetic cluster analysis

Similar to prior work [1], in our cluster analysis we also discovered that OCD and TS belonged to the same group, in line with substantial evidence that the two disorders overlap in many ways that suggest a much closer relationship. It was reported more than third of people with TS had OCD [139–141], leading to the difficulty of telling the difference between the two disorders. This finding is also consistent with the hypothesis that family history studies of comorbidity have found familial aggregation with TS, especially for early-onset OCD, and familial aggregation with other psychiatric disorders such as anxiety [142]. Furthermore, we found that two childhood-onset disorders (i.e., ADHD and ASD) were separately divided into two diverse groups; specifically, ADHD was shown to be genetically similar to PTSD, AD, and MDD, while ASD exhibited more genetic similarity with OCD, TS, BIP, SCZ, and AN, suggesting that the presentation of psychiatric symptoms have a broad spectrum ranging from childhood to adulthood and that the vulnerability for some of psychiatric disorders might begin early in the stage of neurodevelopment [143–145]. Note that, our cluster result may be not completely in agreement with the observation in clinical practice as it was generated based only on genetic similarity.

#### Comparison of gene enrichment analysis

Because of only using a small fraction of lead pleiotropic SNPs associated across psychiatric disorders, prior work only discovered significant enrichment of genes expressed in brain [1]. Besides brain, we additionally showed significant enrichment in pancreas, liver, and heart, offering more insights into the biological processes underlying these disorders. The relationship of psychiatric disorders with brain is well established [3, 144, 146–149], and their connection with liver is supported by the occurrence of various psychiatric syndromes among patents with liver-relevant diseases observed in prior literature [150, 151]. There also exists sufficient evidence supporting its link with liver. For example, the concept of cerebral intoxication by nitrogenous substances derived from the intestine partly accounts for portal-systemic encephalopathy and also offers a rational basis for effective therapy of liver disease [150]. Twin studies and molecular genetic studies further revealed substantial genetic correlation between coronary artery disease and psychiatric disorders such as schizophrenia, bipolar disorder, and major depressive disorder, and even suggested that both may actually cause one another [152]. Moreover, the animal experiment showed that brain-pancreas relative protein exposed to chronic unpredictable mild stress would induce depression in male rats [153], indicating pancreas is a tissue closely related to disease process of psychiatric disorders. As another evidence, it was demonstrated that nearly half of patients with pancreatic carcinoma had evident premorbid psychiatric symptoms [154], indicating their important indicator roles in the diagnosis of pancreatic carcinoma. Finally, both liver and pancreas are metabolism-related organs, implying that psychiatric disorders are associated with metabolic relevant biological process. This finding is consistent with the observation that persons with psychiatric disorders are at increased risk of developing metabolic syndromes, diabetes, and cardiovascular diseases, which has become one of the greatest challenges in psychiatric practice [155–157].

### Important statistical and scientific implications of our findings

Our finding has important implications from both the statistical and scientific perspectives. First, the extensive genetic overlap among psychiatric disorders aids to discover potentially genetic connection that cannot be observed in a single phenotype study, it also promotes the ability to substantially boost statistical power and improve prediction accuracy in joint analysis by borrowing phenotypic correlation across psychiatric traits [13, 158], which means that more fruitful gains would be available by applying cost-effective pleiotropy-informed statistical approaches to mine existing and future data sources of psychiatric disorders. Second, advances in knowledge of common genetic architecture of psychiatric disorders are critical for developing novel genetically based therapeutic strategies. It is very likely that a target treatment designed for one disorder has a broader therapeutic role in other disorders, implying wider patients could possibly benefit from such research [11]. Third, among the pleiotropic associations, we emphasize the so-called antagonistic effect phenomenon that a specific gene may show strong associations with multiple psychiatric disorder but the directions of the genetic effect may be opposite in each other. This finding is particularly important for discovering molecular targets especially intending to repair mutations via genome-editing techniques such as the CRISPR-CAS system, since this might lead to unexpected genetic, and therefore phenotypic, side impacts [17]. Fourth, the positively causal associations between distinct psychiatric disorders offer important insight into the development of prevention and treatment strategies in the clinic. For example, much attention should be paid for a child with ADHD in order to avoid or decrease the risk of other adulthood-onset psychiatric disorders such as AN, BIP, and MDD.

### Potential limitations

Finally, we highlight that our results should be interpreted in consideration with several limitations. First, although our results suggest that, although the comorbidity of psychiatric disorders is partly explained by common genetic components, the shared biological mechanism underlying psychiatric disorders is largely not clear [13, 78], the functional role of these pleiotropic genes remains completely unknown, and the causal mechanism among psychiatric disorders is not yet understood. Therefore, further experimental and methodological investigations are warranted. Second, because rare variants were not available in most GWAS summary data, in the present we only focused on SNPs with MAF > 0.01 and cannot detect shared rare SNPs. Exploring the pleiotropy for rare genetic loci underlying psychiatric disorders can certainly offer more in-depth understanding of shared genetic foundation between these disorders [159].

Third, reproducibility is a well-known important principle in evaluating the findings of association studies [160]; however, at this time, we are unaware of other similar large-scale European-only summary statistics that could be employed for replication of our association discoveries. Again, because of unavailability of relevant data, we primarily focused on our pleiotropy analysis in the European population; therefore, due to the trans-ethnic diversity of genetic architecture in many complex traits including psychiatric disorders [161], it is unclear whether our findings can be transportable to other ancestral groups such as East Asians.

Fourth, the substantial imbalance in sample sizes across these psychiatric disorders (ranging from 9725 for OCD to 480,359 for MDD) resulted in varying power, which might undermine our findings. As the power of identifying pleiotropic associations partly depends on sample size; therefore, larger samples are required for some disorders with small sizes. Fifth, we here only explored disorder-common genetic loci. Understanding disorder-specific associated genes is also equally important and has the potential to elucidate genetic difference between psychiatric disorders [162], to distinguish one particular psychiatric disorder from others and to shed light on the reason why a treatment is effective for only one disorder but is not for others.

## Conclusions

To our knowledge, this study is among the first large-scale effort to characterize the gene-level pleiotropy among a greatly expanded set of psychiatric disorders, and provides important insight into shared genetic etiology underlying these disorders. The findings would inform psychiatric nosology, identify potential neurobiological mechanisms predisposing to specific clinical presentations, and pave the way to effective drug targets for clinical treatment.

## Supplementary Information

**Additional file 1: Table S1.** A selective overview of previous pleiotropy studies on psychiatric disorders.

**Additional file 2: Figure S1-S5. Figure S1.** Graphical framework of the Mendelian randomization method using SNPs as instrumental variables for an exposure. A valid Mendelian randomization requires each of used SNP instruments satisfies three key model assumptions: (1) the relevance assumption, (2) the independence assumption, and (3) the exclusion restriction assumption. In the plot, solid or dotted arrow denotes the presence or absence of directional association. **Figure S2.** (A) Association of horizontal pleiotropy for causal genes, which can be examined by the composite-null based MAIUP method; (B) Association of mediated (or vertical) pleiotropy, which is also known as causality and can be examined by the Mendelian randomization method. **Figure S3.** (A) Number of associated genes (FDR < 0.05) discovered by the maximum *P-*value method between 14 psychiatric disorders; (B) Number of associated genes (FDR < 0.05) discovered by the direct FDR method between 14 psychiatric disorders. **Figure S4.** Result of the LRT method for examining the overall pleiotropy for each pair of the 14 psychiatric disorders. The *P* value is shown in the scale of -log10. Significant pleiotropy is marked with an asterisk after Bonferroni correction. **Figure S5.** (A) Proportion of heterogeneity in SNP genetic effects for pleiotropic genes across all significant pairs of the 14 psychiatric disorders. (B) Correlation of mean proportions of genetic effect heterogeneity of SNPs for pleiotropic genes in each pair of the 14 psychiatric disorders and their cross-trait genetic correlations. The estimated correlation coefficient and *P* value are shown on the top right.

**Additional file 3: Table S2.** Pleiotropic genes for all pairs of 14 psychiatric disorders identified by PLACO (FDR < 0.05).

**Additional file 4: Table S3**. List of all the unique pleiotropic genes and the number of psychiatric disorders which were affected by these genes.

**Additional file 5: Table S4.** Unique genes with drug-gene interaction of directional effects on 14 psychiatric disorders.

**Additional file 6: Table S5.** Results of sensitivity analyses for significantly causal associations between psychiatric disorders.

## Data Availability

All data generated or analyzed during this study are included in this published article and its supplementary information files.

## Abbreviations

AD: Anxiety traits
ADHD: Attention-deficit/hyperactivity trait
AN: Anorexia nervosa
ASD: Autism spectrum trait
AUD: Alcohol use trait
BIP: Bipolar trait
CU: Cannabis use
FDR: False discovery rate
GO: Gene ontology
GWAS: Genome-wide association study
LD: Linkage disequilibrium
LDSC: Linkage disequilibrium score regression
MAF: Minor allele frequency
MAGMA: Multi-marker analysis of genomic annotation
MDD: Major depression trait
OCD: Obsessive-compulsive disorder
PGC: Psychiatric Genomics Consortium
PLACO: Pleiotropic analysis under composite null hypothesis
PTSD: Posttraumatic stress trait
SCZ: Schizophrenia
SNP: Single-nucleotide polymorphism
TS: Tourette’s syndrome
