# Supplementary material for: A comprehensive gene-centric pleiotropic association analysis for 14 psychiatric disorders with GWAS summary statistics
Source: BMC Med. 2021 Dec 13;19:314. doi: 10.1186/s12916-021-02186-z (PMC8667366; doi:10.1186/s12916-021-02186-z)
Supplement: Supplementary file 1 — Additional file 1: Table S1. A selective overview of previous pleiotropy studies on psychiatric disorders. [file 12916_2021_2186_MOESM1_ESM.docx]

**Additional file 1**

Table S1. A selective overview of previous pleiotropy studies on psychiatric disorders

| Id | Year | Disease | Methods | level | Conclusion | Reference |
| --- | --- | --- | --- | --- | --- | --- |
| 1 | 2013 | SCZ and BIP | stratified FDR method, cFDR, and conditional Q-Q plots | SNP | Together, these findings show the feasibility of genetic pleiotropy-informed methods to improve gene discovery in SCZ and BIP and indicate overlapping genetic mechanisms between these two disorders. | [[1](#_ENREF_1)] |
| 2 | 2013 | ADHD, ASD, BIP, MDD and SCZ | meta-analytic approach, polygenic risk-score analysis, pathway analyses and enrichment analysis | SNP | This study identified risk loci with shared effects on five major psychiatric disorders. | [[2](#_ENREF_2)] |
| 3 | 2014 | ADHD, ASD, BIP, MDD and SCZ | GPA | SNP | The GPA method provided substantial evidence for shared genetic architecture underlying psychiatric disorders. | [[3](#_ENREF_3)] |
| 4 | 2014 | SCZ and BIP | gene-gene interaction and generalized multifactor dimensionality reduction (GMDR) method | SNP | It found a positive association between *LMAN2L* (rs6746896) with both BIP and SCZ. It also showed that variants of the *ANK3* gene (rs1938516 and rs10994336) were associated with BIP in Malays and that *LMAN2L* rs2271893 was associated with SCZ in Malays and Indians. | [[4](#_ENREF_4)] |
| 5 | 2014 | SCZ and BIP | sign tests and logistic regression with Bonferroni correction for multiple testing | SNP | This work detected a novel association between suicide attempt and the ITIH3/4-region in a combined group of patients with BIP, SCZ and related psychosis spectrum disorders. It may be useful in understanding molecular mechanisms of suicidal behavior in severe mental disorders. | [[5](#_ENREF_5)] |
| 6 | 2014 | SCZ and BIP | meta-analysis | gene | It found the existence of genetic relationships between schizophrenia, bipolar disorder, and schizoaffective disorder. | [[6](#_ENREF_6)] |
| 7 | 2014 | SCZ and BIP | PRS | SNP | This study indicated that combining diseases with similar genetic risk profiles improved power to detect shared risk loci and that future direct comparison of BIP and SCZ were likely to identify loci with significant differential effects. Identifying these loci should aid in the fundamental understanding of how these diseases differ biologically. The findings also indicated that combining clinical symptom dimensions and polygenic signatures could provide additional information that may someday be used clinically. | [[7](#_ENREF_7)] |
| 8 | 2016 | PTSD, BIP and SCZ | PRS | SNP | This study showed that PTSD had shared polygenic contributions with bipolar disorder and schizophrenia in women | [[8](#_ENREF_8)] |
| 9 | 2016 | SCZ and OCD | logistic regression under an additive model, Gene-based association analysis and polygenic risk scores | SNP | The shared polygenic risk between schizophrenia and OCD may be partially responsible for the frequent comorbidity of both disorders, explaining epidemiological data on cross-disorder risk. This common etiology may have clinical implications. | [[9](#_ENREF_9)] |
| 10 | 2016 | SCZ | cFDR and pathway and network analyses | SNP | Further analyses revealed that these shared genes were enriched in calcium signaling, long-term potentiation and neuroactive ligand receptor interaction pathways that played a critical role in cognitive functions and neuronal plasticity. | [[10](#_ENREF_10)] |
| 11 | 2017 | SCZ, BIP and MDD | chi-square test | SNP | This work implicated that bipolar disorder and major depressive disorder might be subtypes of schizophrenia. | [[11](#_ENREF_11)] |
| 12 | 2017 | ADHD, ASD, BIP, SCZ and AN | SNP-heritability and genetic correlations estimated in GCTA, stratified false discovery rates, and LDSC | SNP | This study indicated shared risk across major psychiatric disorders. | [[12](#_ENREF_12)] |
| 13 | 2017 | ASD, ADHD, OCD and MDD | polygenic risk score, joint categorical-continuous trait twin models | SNP | This study indicated that genetic factors which predisposed to psychiatric disorders were also associated with milder variation in characteristic disorders throughout the general population for many psychiatric phenotypes, and supported the conceptualization of psychiatric disorders as the extreme ends of continuous disorders. | [[13](#_ENREF_13)] |
| 14 | 2017 | ASD and SCZ | meta-analysis, association test, binomial sign test, and LDSC | SNP | This study identified a significant genetic correlation with schizophrenia and association of ASD with several neurodevelopmental-related genes such as *EXT1*, *ASTN2*, *MACROD2*, and *HDAC4*. | [[14](#_ENREF_14)] |
| 15 | 2017 | SCZ, AD and ADHD | polygenic risk scores and meta-analysis | SNP | This study confirmed a common genetic etiology of schizophrenia and developmental psychopathology and showed a stronger shared genetic etiology between schizophrenia and adolescent onset psychopathology. | [[15](#_ENREF_15)] |
| 16 | 2017 | ADHD, ASD, BIP, MDD and SCZ | graph-GPA | SNP | The graph-GPA method provided a powerful approach for prioritizing risk-associated genetic variants for major psychiatric disorders. | [[16](#_ENREF_16)] |
| 17 | 2017 | ASD and ADHD | genetic relationship matrix restricted maximum likelihood estimation, pathway analysis and polygenic scoring analyses | SNP | It found that in the general population genetic etiologies between social-communication difficulties and ADHD symptoms were shared throughout child and adolescent development and may implicate similar biological pathways that co-vary during development. Within both the ASD and the ADHD dimension, population-based disorders were also linked to clinical disorder. | [[17](#_ENREF_17)] |
| 18 | 2018 | MDD and alcohol dependence | PRS | SNP | This study indicated the genetic overlap between alcohol dependence and MDD. | [[18](#_ENREF_18)] |
| 19 | 2018 | ADHD, ASD, SCZ and BIP | clustering analysis, and cFDR | SNP | this work identified brain age gap as a genetically modulated trait that offered a window into shared and distinct mechanisms in different brain disorders. | [[19](#_ENREF_19)] |
| 20 | 2018 | BIP and SCZ | sparse canonical correlation analysis | SNP | The analysis offered the potential to include a larger number of fine-grained systematic descriptors, and to include genetic markers associated with other disorders that were genetically correlated with BIP. | [[20](#_ENREF_20)] |
| 21 | 2018 | BIP and cannabis use | PRS | SNP | This study supported an association between high SZ-PGRS and frequent cannabis use before illness onset in psychosis continuum disorders. | [[21](#_ENREF_21)] |
| 22 | 2018 | CU and SCZ | LDSC | SNP | This study provided new insights into the etiology of cannabis use and its relation with mental health. | [[22](#_ENREF_22)] |
| 23 | 2018 | AN, AD, ASD, BIP, MDD, OCD, PTSD, SCZ and TS | LDSC | SNP | It found evidence for widespread common genetic risk sharing among neurological disorders or across neurological and psychiatric disorders, and it showed that both psychiatric and neurological disorders had robust correlations with cognitive and personality measures. | [[23](#_ENREF_23)] |
| 24 | 2018 | ASD, SCZ, BIP and MDD | LDSC | SNP | It replicated broad transcriptomic and cell type-specific patterns independently for ASD, SCZ, and BIP. | [[24](#_ENREF_24)] |
| 25 | 2018 | SCZ and CU | LDSC | SNP | It indicated a causal influence of schizophrenia on cannabis use and substantial genetic overlap between cannabis use and use of other substances, mental health, and personality disorders, such as risk-taking and extraversion. | [[25](#_ENREF_25)] |
| 26 | 2018 | ASD and SCZ | LDSC, polygenic risk scores, Mixed Poisson regression, GCTA, and Attrition analysis in ALSPAC | SNP | This work discovered that clinical ASD and schizophrenia shared some genetic influences with impairments in social communication, but revealed distinct developmental profiles in their genetic links, consistent with the onset of clinical symptoms. | [[26](#_ENREF_26)] |
| 27 | 2018 | SCZ, PTSD, BIP and MDD | SNP-chip heritability estimation with GCTA, Polygenic risk scoring, meta-analyses and LDSC | SNP | This work demonstrated genetic influences on the development of PTSD, identified shared genetic risk between PTSD and other psychiatric disorders. | [[27](#_ENREF_27)] |
| 28 | 2019 | ADHD, ASD, BIP, MDD and SCZ | PRS | SNP | This work suggested that the molecular genetic factors contributing to variability in executive function during typical development were at least partially overlapping with those associated with psychiatric disorders. | [[28](#_ENREF_28)] |
| 29 | 2020 | AN and OCD | LDSC | SNP | This study confirmed and extended genetic epidemiological findings of shared risk between AN and OCD. | [[29](#_ENREF_29)] |
| 30 | 2020 | SCZ, BIP and ADHD | cFDR | SNP | This review provided a methodological comparison between cFDR and other relevant cross-trait analytical tools and demonstrated how cFDR analysis may provide novel insights into the genetic relationship between complex phenotypes such as major psychiatric disorders. | [[30](#_ENREF_30)] |
| 31 | 2020 | SCZ, BIP and MDD | cFDR | SNP | Extensive polygenic overlap between BMI and SCZ, BIP, and MDD were found, and 111 shared genetic loci were identified, implicating novel functional mechanisms. | [[31](#_ENREF_31)] |
| 32 | 2020 | AD, ASD, ADHD, MDD, BIP, SCZ, AN, OCD and PTSD | factor score models | SNP | This work suggested that common genetic variants underlying the risk of anxiety disorders contributed to elevated risks of MDD, SCZ, ADHD and neuroticism and reduced quality of life, putamen volume and cognitive performance, and that the comorbidity of anxiety disorders was partly explained by common genetic variants. | [[32](#_ENREF_32)] |
| 33 | 2021 | SCZ, BIP, MDD, ADHD, AN, ASD, OCD and TS | CC-GWAS | SNP | This study robustly identified loci with different allele frequencies among cases of different disorders. | [[33](#_ENREF_33)] |
| 34 | 2021 | SCZ and BIP | LDSC, cFDR | SNP | This study provided evidence of shared genetic architecture of schizophrenia, bipolar disorder, and lifespan. | [[34](#_ENREF_34)] |

**References**

1. Andreassen OA, Thompson WK, Schork AJ, Ripke S, Mattingsdal M, Kelsoe JR, Kendler KS, O'Donovan MC, Rujescu D, Werge T: **Improved detection of common variants associated with schizophrenia and bipolar disorder using pleiotropy-informed conditional false discovery rate**. *PLoS Genet* 2013, **9**(4):e1003455.

2. Consortium C-DGotPG: **Identification of risk loci with shared effects on five major psychiatric disorders: a genome-wide analysis**. *The Lancet* 2013, **381**(9875):1371-1379.

3. Chung D, Yang C, Li C, Gelernter J, Zhao H: **GPA: a statistical approach to prioritizing GWAS results by integrating pleiotropy and annotation**. *PLoS Genet* 2014, **10**(11):e1004787.

4. Lim CH, Zain SM, Reynolds GP, Zain MA, Roffeei SN, Zainal NZ, Kanagasundram S, Mohamed Z: **Genetic association of LMAN2L gene in schizophrenia and bipolar disorder and its interaction with ANK3 gene polymorphism**. *Progress in Neuro-Psychopharmacology and Biological Psychiatry* 2014, **54**:157-162.

5. Finseth PI, Sønderby IE, Djurovic S, Agartz I, Malt UF, Melle I, Morken G, Andreassen OA, Vaaler AE, Tesli M: **Association analysis between suicidal behaviour and candidate genes of bipolar disorder and schizophrenia**. *Journal of affective disorders* 2014, **163**:110-114.

6. Cardno AG, Owen MJ: **Genetic relationships between schizophrenia, bipolar disorder, and schizoaffective disorder**. *Schizophrenia bulletin* 2014, **40**(3):504-515.

7. Ruderfer DM, Fanous AH, Ripke S, McQuillin A, Amdur RL, Gejman PV, O'Donovan MC, Andreassen OA, Djurovic S, Hultman CM: **Polygenic dissection of diagnosis and clinical dimensions of bipolar disorder and schizophrenia**. *Molecular psychiatry* 2014, **19**(9):1017-1024.

8. Sumner JA, Duncan L, Ratanatharathorn A, Roberts AL, Koenen KC: **PTSD has shared polygenic contributions with bipolar disorder and schizophrenia in women**. *Psychological medicine* 2016, **46**(3):669-671.

9. Costas J, Carrera N, Alonso P, Gurriarán X, Segalàs C, Real E, López-Solà C, Mas S, Gassó P, Domènech L: **Exon-focused genome-wide association study of obsessive-compulsive disorder and shared polygenic risk with schizophrenia**. *Translational psychiatry* 2016, **6**(3):e768-e768.

10. Chen J, Bacanu S-A, Yu H, Zhao Z, Jia P, Kendler KS, Kranzler HR, Gelernter J, Farrer L, Minica C: **Genetic relationship between schizophrenia and nicotine dependence**. *Scientific reports* 2016, **6**(1):1-10.

11. Chen X, Long F, Cai B, Chen X, Qin L, Chen G: **A novel relationship for schizophrenia, bipolar, and major depressive disorder. Part 8: a hint from chromosome 8 high density association screen**. *Molecular neurobiology* 2017, **54**(8):5868-5882.

12. Schork AJ, Won H, Appadurai V, Nudel R, Gandal M, Delaneau O, Hougaard DM, Bækved-Hansen M, Bybjerg-Grauholm J, Pedersen MG: **A genome-wide association study for shared risk across major psychiatric disorders in a nation-wide birth cohort implicates fetal neurodevelopment as a key mediator**. *bioRxiv* 2017:240911.

13. Taylor MJ, Martin J, Lu Y, Brikell I, Lundström S, Larsson H, Lichtenstein P: **Genetic evidence for shared risks across psychiatric disorders and related traits in a Swedish population twin sample**. *BioRxiv* 2017:234963.

14. Consortium TASDWGoTPG: **Meta-analysis of GWAS of over 16,000 individuals with autism spectrum disorder highlights a novel locus at 10q24. 32 and a significant overlap with schizophrenia**. *Molecular autism* 2017, **8**:1-17.

15. Nivard MG, Gage SH, Hottenga JJ, Van Beijsterveldt CE, Abdellaoui A, Bartels M, Baselmans BM, Ligthart L, Pourcain BS, Boomsma DI: **Genetic overlap between schizophrenia and developmental psychopathology: longitudinal and multivariate polygenic risk prediction of common psychiatric traits during development**. *Schizophrenia Bulletin* 2017, **43**(6):1197-1207.

16. Chung D, Kim HJ, Zhao H: **graph-GPA: a graphical model for prioritizing GWAS results and investigating pleiotropic architecture**. *PLoS computational biology* 2017, **13**(2):e1005388.

17. Stergiakouli E, Smith GD, Martin J, Skuse DH, Viechtbauer W, Ring SM, Ronald A, Evans DE, Fisher SE, Thapar A: **Shared genetic influences between dimensional ASD and ADHD symptoms during child and adolescent development**. *Molecular Autism* 2017, **8**(1):1-13.

18. Foo JC, Streit F, Treutlein J, Ripke S, Witt SH, Strohmaier J, Degenhardt F, Forstner AJ, Hoffmann P, Soyka M: **Shared genetic etiology between alcohol dependence and major depressive disorder**. *Psychiatric genetics* 2018, **28**(4):66.

19. Kaufmann T, van der Meer D, Doan NT, Schwarz E, Lund MJ, Agartz I, Alnæs D, Barch DM, Baur-Streubel R, Bertolino A: **Genetics of brain age suggest an overlap with common brain disorders**. *BioRxiv* 2018:303164.

20. Leonenko G, Di Florio A, Allardyce J, Forty L, Knott S, Jones L, Gordon‐Smith K, Owen MJ, Jones I, Walters J: **A data‐driven investigation of relationships between bipolar psychotic symptoms and schizophrenia genome‐wide significant genetic loci**. *American Journal of Medical Genetics Part B: Neuropsychiatric Genetics* 2018, **177**(4):468-475.

21. Aas M, Melle I, Bettella F, Djurovic S, Le Hellard S, Bjella T, Ringen PA, Lagerberg TV, Smeland OB, Agartz I: **Psychotic patients who used cannabis frequently before illness onset have higher genetic predisposition to schizophrenia than those who did not**. *Psychological medicine* 2018, **48**(1):43-49.

22. Pasman JA, Verweij KJ, Gerring Z, Stringer S, Sanchez-Roige S, Treur JL, Abdellaoui A, Nivard MG, Baselmans BM, Ong J-S: **GWAS of lifetime cannabis use reveals new risk loci, genetic overlap with psychiatric traits, and a causal effect of schizophrenia liability**. *Nature neuroscience* 2018, **21**(9):1161-1170.

23. Anttila V, Bulik-Sullivan B, Finucane HK, Walters RK, Bras J, Duncan L, Escott-Price V, Falcone GJ, Gormley P, Malik R: **Analysis of shared heritability in common disorders of the brain**. *Science* 2018, **360**(6395).

24. Gandal MJ, Haney JR, Parikshak NN, Leppa V, Ramaswami G, Hartl C, Schork AJ, Appadurai V, Buil A, Werge TM: **Shared molecular neuropathology across major psychiatric disorders parallels polygenic overlap**. *Science* 2018, **359**(6376):693-697.

25. Pasman JA, Verweij KJ, Gerring Z, Stringer S, Sanchez-Roige S, Treur JL, Abdellaoui A, Nivard MG, Baselmans BM, Ong J-S: **Genome-wide association analysis of lifetime cannabis use (N= 184,765) identifies new risk loci, genetic overlap with mental health, and a causal influence of schizophrenia on cannabis use**. *bioRxiv* 2018:234294.

26. St Pourcain B, Robinson EB, Anttila V, Sullivan BB, Maller J, Golding J, Skuse D, Ring S, Evans DM, Zammit S: **ASD and schizophrenia show distinct developmental profiles in common genetic overlap with population-based social communication difficulties**. *Molecular psychiatry* 2018, **23**(2):263-270.

27. Duncan LE, Ratanatharathorn A, Aiello AE, Almli LM, Amstadter AB, Ashley-Koch AE, Baker DG, Beckham JC, Bierut LJ, Bisson J: **Largest GWAS of PTSD (N= 20 070) yields genetic overlap with schizophrenia and sex differences in heritability**. *Molecular psychiatry* 2018, **23**(3):666-673.

28. Schork A, Brown T, Hagler D, Thompson W, Chen CH, Dale A, Jernigan T, Akshoomoff N, Pediatric Imaging N, Study G: **Polygenic risk for psychiatric disorders correlates with executive function in typical development**. *Genes, Brain and Behavior* 2019, **18**(4):e12480.

29. Yilmaz Z, Halvorsen M, Bryois J, Yu D, Thornton LM, Zerwas S, Micali N, Moessner R, Burton CL, Zai G: **Examination of the shared genetic basis of anorexia nervosa and obsessive–compulsive disorder**. *Molecular psychiatry* 2020, **25**(9):2036-2046.

30. Smeland OB, Frei O, Shadrin A, O’Connell K, Fan C-C, Bahrami S, Holland D, Djurovic S, Thompson WK, Dale AM: **Discovery of shared genomic loci using the conditional false discovery rate approach**. *Human genetics* 2020, **139**(1):85-94.

31. Bahrami S, Steen NE, Shadrin A, O’Connell K, Frei O, Bettella F, Wirgenes KV, Krull F, Fan CC, Dale AM: **Shared genetic loci between body mass index and major psychiatric disorders: a genome-wide association study**. *JAMA psychiatry* 2020, **77**(5):503-512.

32. Ohi K, Otowa T, Shimada M, Sasaki T, Tanii H: **Shared genetic etiology between anxiety disorders and psychiatric and related intermediate phenotypes**. *Psychological medicine* 2020, **50**(4):692-704.

33. Peyrot WJ, Price AL: **Identifying loci with different allele frequencies among cases of eight psychiatric disorders using CC-GWAS**. *Nature genetics* 2021, **53**(4):445-454.

34. Muntané G, Farré X, Bosch E, Martorell L, Navarro A, Vilella E: **The shared genetic architecture of schizophrenia, bipolar disorder and lifespan**. *Human Genetics* 2021, **140**(3):441-455.
