# Supplementary material for: A comprehensive gene-centric pleiotropic association analysis for 14 psychiatric disorders with GWAS summary statistics
Source: BMC Med. 2021 Dec 13;19:314. doi: 10.1186/s12916-021-02186-z (PMC8667366; doi:10.1186/s12916-021-02186-z)
Supplement: Supplementary file 2 — Additional file 2: Figure S1-S5. Figure S1. Graphical framework of the Mendelian randomization method using SNPs as instrumental variables for an exposure. A valid Mendelian randomization requires each of used SNP instruments satisfies three key model assumptions: (1) the relevance assumption, (2) the independence assumption, and (3) the exclusion restriction assumption. In the plot, solid or dotted arrow denotes the presence or absence of directional association. Figure S2. (A) Association of horizontal pleiotropy for causal genes, which can be examined by the composite-null based MAIUP method; (B) Association of mediated (or vertical) pleiotropy, which is also known as causality and can be examined by the Mendelian randomization method. Figure S3. (A) Number of associated genes (FDR < 0.05) discovered by the maximum P-value method between 14 psychiatric disorders; (B) Number of associated genes (FDR < 0.05) discovered by the direct FDR method between 14 psychiatric disorders. Figure S4. Result of the LRT method for examining the overall pleiotropy for each pair of the 14 psychiatric disorders. The P value is shown in the scale of -log10. Significant pleiotropy is marked with an asterisk after Bonferroni correction. Figure S5. (A) Proportion of heterogeneity in SNP genetic effects for pleiotropic genes across all significant pairs of the 14 psychiatric disorders. (B) Correlation of mean proportions of genetic effect heterogeneity of SNPs for pleiotropic genes in each pair of the 14 psychiatric disorders and their cross-trait genetic correlations. The estimated correlation coefficient and P value are shown on the top right. [file 12916_2021_2186_MOESM2_ESM.docx]

**Additional file 2**


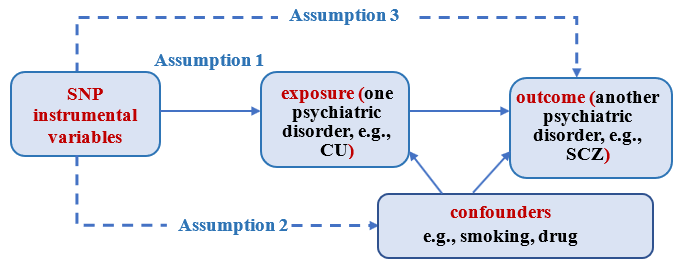


Figure S1. Graphical framework of the Mendelian randomization method using SNPs as instrumental variables for an exposure. A valid Mendelian randomization requires each of used SNP instruments satisfies three key model assumptions: (1) the relevance assumption, (2) the independence assumption, and (3) the exclusion restriction assumption. In the plot, solid or dotted arrow denotes the presence or absence of directional association.


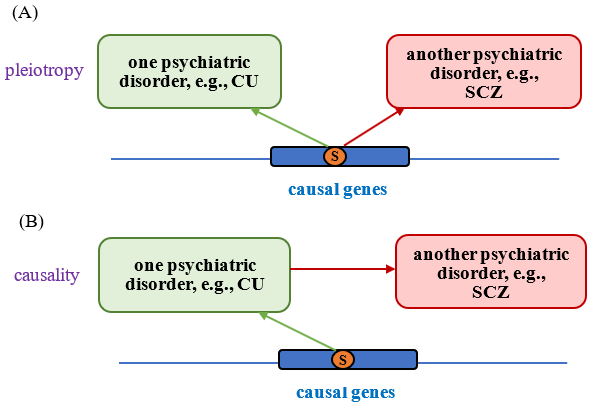


Figure S2. (A) Association of horizontal pleiotropy for causal genes, which can be examined by the composite-null based MAIUP method; (B) Association of mediated (or vertical) pleiotropy, which is also known as causality and can be examined by the Mendelian randomization method.


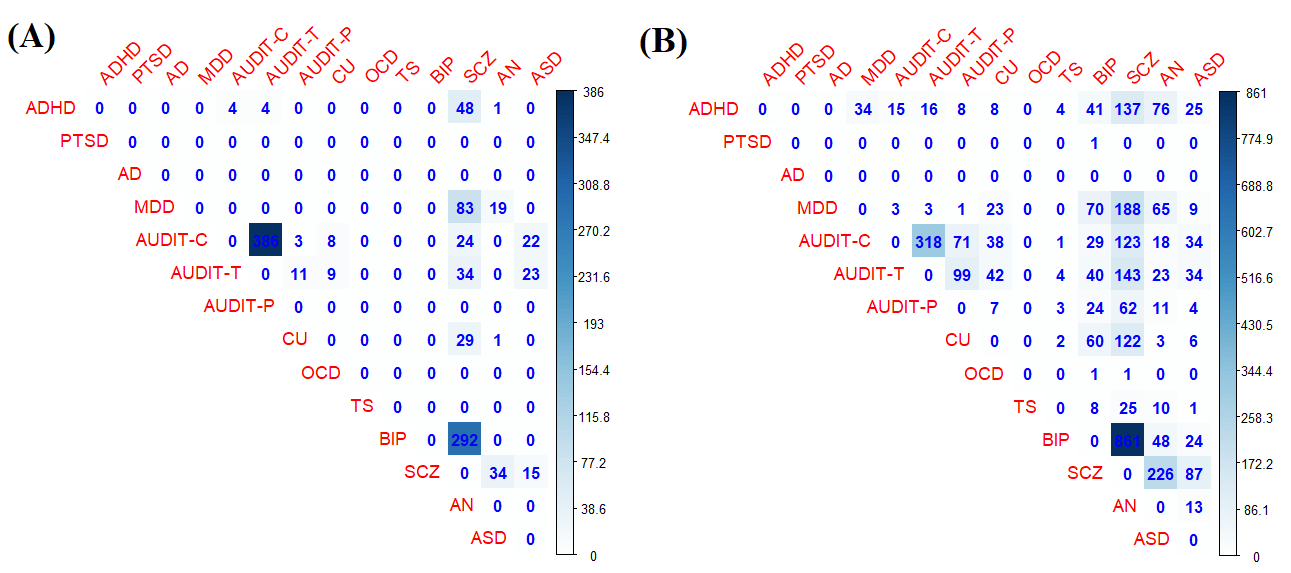


Figure S3. (A) Number of associated genes (FDR<0.05) discovered by the maximum *P-*value method between 14 psychiatric disorders; (B) Number of associated genes (FDR<0.05) discovered by the direct FDR method between 14 psychiatric disorders.


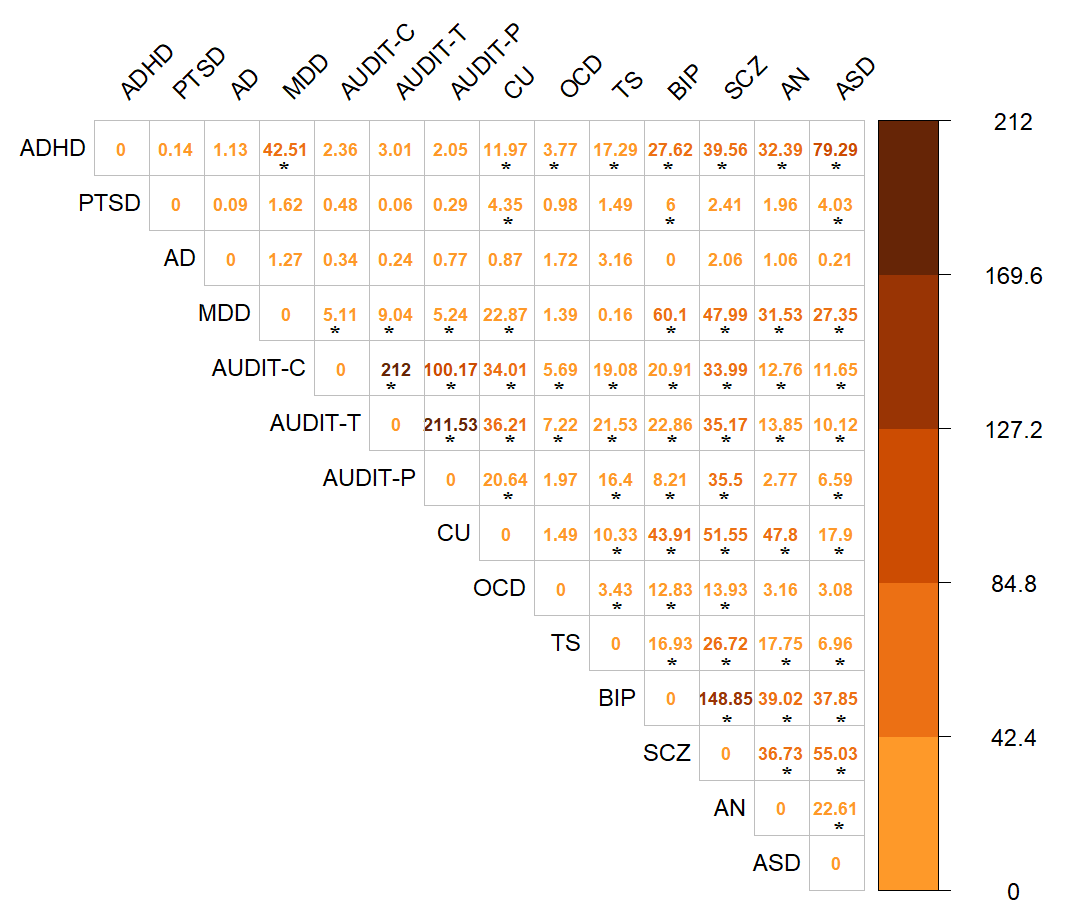


Figure S4. Result of the LRT method for examining the overall pleiotropy for each pair of the 14 psychiatric disorders. The *P* value is shown in the scale of -log10. Significant pleiotropy is marked with an asterisk after Bonferroni correction.


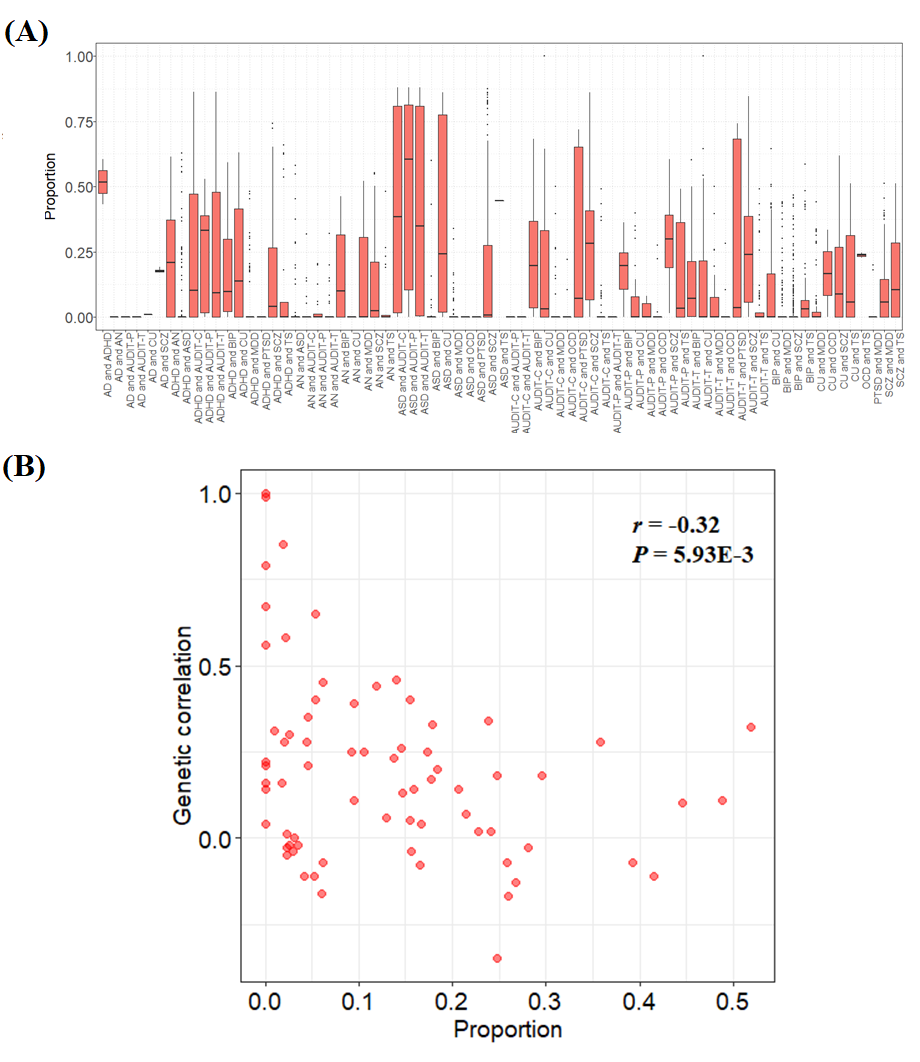


Figure S5. (A) Proportion of heterogeneity in SNP genetic effects for pleiotropic genes across all significant pairs of the 14 psychiatric disorders. (B) Correlation of mean proportions of genetic effect heterogeneity of SNPs for pleiotropic genes in each pair of the 14 psychiatric disorders and their cross-trait genetic correlations. The estimated correlation coefficient and *P* value are shown on the top right.
