# Supplementary material for: A comprehensive gene-centric pleiotropic association analysis for 14 psychiatric disorders with GWAS summary statistics
Source: BMC Med. 2021 Dec 13;19:314. doi: 10.1186/s12916-021-02186-z (PMC8667366; doi:10.1186/s12916-021-02186-z)
Supplement: Supplementary file 6 — Additional file 6: Table S5. Results of sensitivity analyses for significantly causal associations between psychiatric disorders. [file 12916_2021_2186_MOESM6_ESM.docx]

**Additional file 6**

Table S5. Results of sensitivity analyses for significantly causal associations between psychiatric disorders

| one  disorder | another  disorder | weighted median method | | | |  | Egger regression | | | |  | Egger intercept | | | |
| --- | --- | --- | --- | --- | --- | --- | --- | --- | --- | --- | --- | --- | --- | --- | --- |
|  |  | beta | lower | upper | *P* |  | beta | lower | upper | *P* |  | intercept | lower | upper | *P* |
| AN2 | BIP | 0.10 | 0.03 | 0.16 | 4.79E-03 |  | 0.14 | -0.04 | 0.31 | 1.25E-01 |  | -0.005 | -0.020 | 0.010 | 0.517 |
| AN2 | MDD | 0.08 | 0.03 | 0.12 | 7.34E-04 |  | 0.06 | -0.05 | 0.18 | 2.72E-01 |  | 0.002 | -0.007 | 0.012 | 0.644 |
| AN2 | OCD | 0.28 | 0.12 | 0.44 | 5.13E-04 |  | 0.11 | -0.24 | 0.46 | 5.38E-01 |  | 0.011 | -0.019 | 0.040 | 0.474 |
| AN2 | PTSD | 0.10 | 0.04 | 0.17 | 2.85E-03 |  | 0.00 | -0.17 | 0.16 | 9.57E-01 |  | 0.007 | -0.007 | 0.020 | 0.315 |
| AN2 | SCZ | 0.10 | 0.04 | 0.15 | 3.89E-04 |  | 0.13 | -0.04 | 0.31 | 1.40E-01 |  | -0.001 | -0.016 | 0.014 | 0.913 |
| AN2 | TS | 0.13 | 0.00 | 0.25 | 4.92E-02 |  | 0.32 | -0.02 | 0.65 | 6.16E-02 |  | -0.017 | -0.045 | 0.011 | 0.236 |
| AUDIT_C | Cannabis | 1.24 | 0.47 | 2.01 | 1.55E-03 |  | 0.31 | -2.10 | 2.71 | 7.99E-01 |  | 0.011 | -0.005 | 0.028 | 0.166 |
| AUDIT_C | PTSD | -1.57 | -2.61 | -0.53 | 3.16E-03 |  | -2.34 | -4.19 | -0.48 | 1.35E-02 |  | 0.008 | -0.005 | 0.021 | 0.213 |
| AUDIT_C | TS | -2.04 | -4.02 | -0.07 | 4.23E-02 |  | 0.21 | -2.88 | 3.31 | 8.91E-01 |  | -0.020 | -0.044 | 0.004 | 0.101 |
| BIP | Cannabis | 0.07 | 0.03 | 0.10 | 2.31E-04 |  | 0.02 | -0.10 | 0.13 | 7.82E-01 |  | 0.004 | -0.005 | 0.013 | 0.373 |
| BIP | MDD | 0.08 | 0.04 | 0.11 | 1.05E-05 |  | 0.05 | -0.07 | 0.16 | 4.31E-01 |  | 0.004 | -0.005 | 0.014 | 0.353 |
| BIP | OCD | 0.19 | 0.07 | 0.31 | 1.49E-03 |  | 0.29 | -0.06 | 0.63 | 9.96E-02 |  | -0.010 | -0.037 | 0.018 | 0.499 |
| BIP | PTSD | 0.08 | 0.03 | 0.13 | 9.62E-04 |  | 0.13 | -0.01 | 0.27 | 5.97E-02 |  | -0.004 | -0.015 | 0.008 | 0.525 |
| BIP | SCZ | 0.36 | 0.31 | 0.40 | 2.16E-54 |  | 0.13 | -0.03 | 0.30 | 1.05E-01 |  | 0.020 | 0.006 | 0.033 | 0.004 |
| Cannabis | AN2 | 0.14 | 0.02 | 0.25 | 1.93E-02 |  | 0.13 | -0.13 | 0.38 | 3.26E-01 |  | 0.001 | -0.014 | 0.016 | 0.925 |
| Cannabis | AUDIT_C | 0.01 | 0.00 | 0.02 | 4.58E-03 |  | 0.01 | -0.01 | 0.03 | 2.28E-01 |  | 0.001 | 0.000 | 0.002 | 0.259 |
| Cannabis | PTSD | 0.03 | -0.07 | 0.14 | 5.45E-01 |  | -0.07 | -0.23 | 0.09 | 3.99E-01 |  | 0.011 | 0.000 | 0.021 | 0.043 |
| MDD | AD | 0.65 | 0.36 | 0.94 | 1.22E-05 |  | 0.88 | -0.18 | 1.95 | 1.03E-01 |  | -0.015 | -0.062 | 0.032 | 0.538 |
| MDD | AN2 | 0.38 | 0.24 | 0.52 | 6.41E-08 |  | 0.40 | -0.04 | 0.83 | 7.48E-02 |  | -0.001 | -0.022 | 0.020 | 0.901 |
| MDD | BIP | 0.16 | 0.04 | 0.29 | 8.06E-03 |  | 0.23 | 0.00 | 0.46 | 5.23E-02 |  | -0.001 | -0.012 | 0.011 | 0.922 |
| MDD | PTSD | 0.30 | 0.17 | 0.43 | 1.11E-05 |  | 0.13 | -0.13 | 0.39 | 3.31E-01 |  | 0.008 | -0.005 | 0.022 | 0.216 |
| MDD | SCZ | 0.28 | 0.18 | 0.39 | 5.89E-08 |  | 0.23 | -0.08 | 0.53 | 1.46E-01 |  | 0.008 | -0.008 | 0.024 | 0.327 |
| OCD | BIP | 0.04 | -0.01 | 0.09 | 1.48E-01 |  | 0.02 | -0.09 | 0.12 | 7.44E-01 |  | 0.008 | -0.017 | 0.034 | 0.510 |
| OCD | TS | 0.16 | 0.06 | 0.26 | 2.15E-03 |  | 0.10 | -0.10 | 0.29 | 3.30E-01 |  | 0.017 | -0.026 | 0.060 | 0.436 |
| PTSD | AN2 | 0.10 | -0.01 | 0.22 | 7.51E-02 |  | 0.09 | -0.12 | 0.30 | 3.66E-01 |  | 0.000 | -0.022 | 0.023 | 0.966 |
| SCZ | AD | 0.11 | 0.03 | 0.19 | 5.14E-03 |  | 0.24 | 0.02 | 0.47 | 3.36E-02 |  | -0.011 | -0.026 | 0.004 | 0.145 |
| SCZ | AN2 | 0.11 | 0.08 | 0.15 | 2.21E-09 |  | 0.08 | -0.03 | 0.20 | 1.51E-01 |  | 0.001 | -0.006 | 0.009 | 0.731 |
| SCZ | BIP | 0.43 | 0.39 | 0.46 | 2.76E-108 |  | 0.33 | 0.23 | 0.43 | 4.92E-10 |  | 0.006 | -0.001 | 0.013 | 0.100 |
| SCZ | Cannabis | 0.08 | 0.05 | 0.10 | 2.00E-08 |  | 0.07 | -0.01 | 0.15 | 6.72E-02 |  | 0.000 | -0.006 | 0.005 | 0.934 |
| SCZ | MDD | 0.10 | 0.07 | 0.12 | 2.96E-13 |  | 0.11 | 0.03 | 0.19 | 7.60E-03 |  | -0.001 | -0.007 | 0.004 | 0.662 |
| SCZ | OCD | 0.20 | 0.11 | 0.29 | 1.27E-05 |  | -0.02 | -0.26 | 0.23 | 8.84E-01 |  | 0.016 | -0.001 | 0.032 | 0.068 |
| SCZ | PTSD | 0.08 | 0.05 | 0.12 | 9.31E-06 |  | 0.12 | 0.02 | 0.21 | 1.57E-02 |  | -0.002 | -0.009 | 0.004 | 0.474 |
| SCZ | TS | 0.06 | -0.01 | 0.13 | 1.01E-01 |  | 0.08 | -0.10 | 0.27 | 3.80E-01 |  | -0.001 | -0.014 | 0.012 | 0.888 |
| TS | OCD | 0.23 | 0.09 | 0.38 | 1.21E-03 |  | 0.17 | -0.11 | 0.45 | 2.35E-01 |  | 0.011 | -0.036 | 0.058 | 0.635 |
| ADHD | AN2 | 0.08 | 0.01 | 0.15 | 1.72E-02 |  | 0.19 | -0.02 | 0.40 | 8.01E-02 |  | -0.007 | -0.025 | 0.010 | 0.387 |
| ADHD | BIP | 0.06 | 0.00 | 0.12 | 5.41E-02 |  | 0.06 | -0.10 | 0.23 | 4.55E-01 |  | 0.001 | -0.013 | 0.014 | 0.924 |
| ADHD | Cannabis | 0.06 | 0.02 | 0.11 | 4.48E-03 |  | 0.01 | -0.10 | 0.12 | 8.52E-01 |  | 0.004 | -0.005 | 0.013 | 0.383 |
| ADHD | MDD | 0.15 | 0.10 | 0.19 | 2.48E-10 |  | 0.23 | 0.11 | 0.36 | 3.00E-04 |  | -0.008 | -0.017 | 0.002 | 0.130 |
| ADHD | PTSD | 0.15 | 0.08 | 0.21 | 1.87E-05 |  | 0.03 | -0.15 | 0.20 | 7.71E-01 |  | 0.007 | -0.007 | 0.022 | 0.334 |
| ADHD | SCZ | 0.09 | 0.04 | 0.14 | 7.39E-04 |  | 0.00 | -0.17 | 0.17 | 9.74E-01 |  | 0.006 | -0.008 | 0.020 | 0.430 |
| ADHD | TS | 0.15 | 0.03 | 0.27 | 1.47E-02 |  | 0.11 | -0.22 | 0.44 | 5.01E-01 |  | 0.009 | -0.018 | 0.036 | 0.523 |
| ASD | Cannabis | 0.04 | -0.02 | 0.10 | 1.70E-01 |  | 0.00 | -0.11 | 0.11 | 9.79E-01 |  | 0.005 | -0.005 | 0.015 | 0.299 |
| ASD | PTSD | 0.10 | 0.02 | 0.19 | 2.09E-02 |  | 0.11 | -0.08 | 0.30 | 2.55E-01 |  | 0.000 | -0.017 | 0.017 | 0.994 |
